# An enduring in vitro wound healing phase recipient by bioactive glass-graphene oxide nanocomposites

**DOI:** 10.1038/s41598-022-20575-z

**Published:** 2022-09-28

**Authors:** Manjubaashini Nandhakumar, Daniel Thangadurai Thangaian, Senthilarasu Sundaram, Anurag Roy, Balakumar Subramanian

**Affiliations:** 1 National Centre for Nanoscience and Nanotechnology, University of Madras, Chennai, 600 025 India; 2 Department of Chemistry and Centre for Nanoscience and Technology, KPR Institute of Engineering and Technology, Coimbatore, 641 407 India; 3 Electrical and Electronics Engineering, School of Engineering and the Built Environment, Edinburgh Napier University, Edinburgh, EH10 5DT UK; 4 Environment and Sustainability Institute, University of Exeter, Penryn, TR10 9FE, UK

**Keywords:** Biological techniques, Biotechnology, Health care

## Abstract

Bioactive glass (BG) is an interesting topic in soft tissue engineering because of its biocompatibility and bonding potential to increase fibroblast cell proliferation, synthesize growth factors, and stimulate granulation tissue development. The proposed BG with and without sodium (Na), prepared by the sol–gel method, is employed in wound healing studies. The BG/graphene oxide (GO) and BG (Na-free)/GO nanocomposites were investigated against fibroblast L929 cells in vitro; the 45S5 BG nanocomposites exhibited desired cell viability (80%), cell proliferation (30%), cell migration (25%), metabolic activity, and wound contraction due to extracellular matrix (ECM) production and enhanced protein release by fibroblast cells. Additionally, the antioxidant assays for BG, BG (Na-free), GO, and BG/GO, BG (Na-free)/GO were evaluated for effective wound healing properties. The results showed decreased inflammation sites in the wound area, assessed by the (2,2-diphenyl-1-picryl-hydrazyl-hydrate) (DPPH) assay with ~ 80% radical scavenging activity, confirming their anti-inflammatory and improved wound healing properties.

## Introduction

The primary research topic in advanced medical bioengineering concerns wound dressing without scarring and other complications. Chronic diabetic wounds are more dangerous than regular^1^ and have a low level of vascular endothelial growth factor (VEGF), resulting in reduced angiogenesis. The danger of tissue necrosis is severe in diabetic people^2^. In the medical profession, treating chronic wounds has become difficult; numerous clinical studies are underway to treat diabetic wounds. As a result, the wound healing material must be partly hydrophilic or hydrophobic, non-toxic, non-allergic, anti-bacterial, allow for oxygen and moisture content, operate as a microbe barrier, and remove excess exudates^3^.

Soft tissue engineering has established a promising wound healing solution with its enhanced healing process and appropriate biocompatible materials, including biopolymer, bioglass (BG), and carbon-based materials^4^. The BG’s substantial properties such as the sustainable release of ions, biocompatibility, anti-bacterial and angiogenic property, and bioactivity are the primary influence of the significant wound healing process in soft tissue regeneration^5,6^. Skin wound healing is a complicated process involving several cell types and processes, including epidermal, fibroblastic, and endothelial cell proliferation, cell migration, extracellular matrix (ECM) formation, and wound contraction, all of which are controlled by various cytokines and growth factors^7,8^. Any skin injury can allow bacteria to readily enter and establish colonies, resulting in serious wound infections^9^. The three-dimensional (3D) non-periodic matrix structure is maintained in the BG with different compositions^10^. For example, 45 wt% SiO_2_, 24.5 wt% CaO, 24.5 wt% Na_2_O, and 6.0 wt% P_2_O_5_ are present in 45S5 BG. Silica is a network former, whereas Ca, P, and Na ions serve as network modifiers^11^. The presence of Si helps precipitation or reconstruction of the surface by non-bridging oxygen sites^12^. It thus improves the attachment of other possible metal ions and other corresponding functional groups for improved bone-bonding ability and mechanical stability^13^. Because of these characteristics, BGs are more suited to soft tissue engineering than hard tissue engineering or implants^14^. The real benefit of BGs in soft tissue engineering is that they have a high level of bioactivity in physiological conditions and a high surface reactivity for producing hydroxyl carbonate apatite (HCA) layers on soft tissue^15^. Despite this, the BGs were widely employed in soft tissue engineering for wound healing applications because of their osteogenic, angiogenesis, proliferators, and biocompatibility properties^16^.

Due to their exceptional mechanical properties and biocompatibility, nano-featured carbonaceous structures such as one-dimensional (1D) carbon nanotubes (CNTs), two-dimensional (2D) layers such as graphene, graphene oxide (GO), and reduced graphene oxide (rGO) are recognized as highly potential materials for reinforcing matrices in biomaterials^17^. In graphene biological research, graphene derivatives and their interactions with bio-organisms have recently received a lot of interest^18^. The use of graphene derivatives for soft tissue engineering is the current trend for quicker and more efficient wound healing^19^. The GO structure has a lot of functional groups that may covalently connect to proteins and growth factors to help cells grow and differentiate faster. GO is prone to surface modification and may be composited with biomaterials to improve its characteristics for various tissue engineering applications^20^. Cell behavior is influenced by the quantitative oxygen available on the GO surface, and a partial decrease in rGO can promote cell adhesion and proliferation^21^. It may also be used to promote wound healing, increase wound contraction, and reduce scar formation^22^. In addition, the wound healing process is categorized into four stages: hemostasis (blood clotting), inflammation (swelling), proliferation (cell multiplication), and tissue remodeling.

Physicochemical and biological analytical techniques were used to investigate and assess the characteristics of 45S5 BG, GO, and their nanocomposites for the distinct stages of wound healing. As a result, BG and GO were chosen as the primary wound-healing materials for chronic and diabetic wounds in the present work based on their merits and abilities. The current report focuses on the importance of Na, which governs the wound healing properties of BG. Because the BG with Na ion may draw down beneficial wound healing qualities like hemostat, the Na may also easily leak out the BG matrix^23^. This is the first report on comprehensive in vitro wound healing for BG and BG (Na-free), which exhibits appreciable scavenging activity at lower concentrations and may exhibit an anti-inflammatory function.

## Results and discussion

The mineralization mechanism for bone growth via calcium and phosphate deposition on the bone matrix will influence bone-forming properties^24^. Mineralization in soft tissues is called ECM production or metastatic categorization in necrotic cells^25,26^. The BG, BG (Na-free), GO and BG/GO, BG (Na-free)/GO nanocomposites were immersed in Simulated Blood Fluid (SBF) solution for up to 28 days to identify the physiochemical pathway of mineral deposition on the surface along with the ECM forming potential of BG in vitro. Later, the samples were collected and dried at predetermined intervals (1st, 14th, and 28th day) for analysis.

### Structural analysis

**Figure 1:**
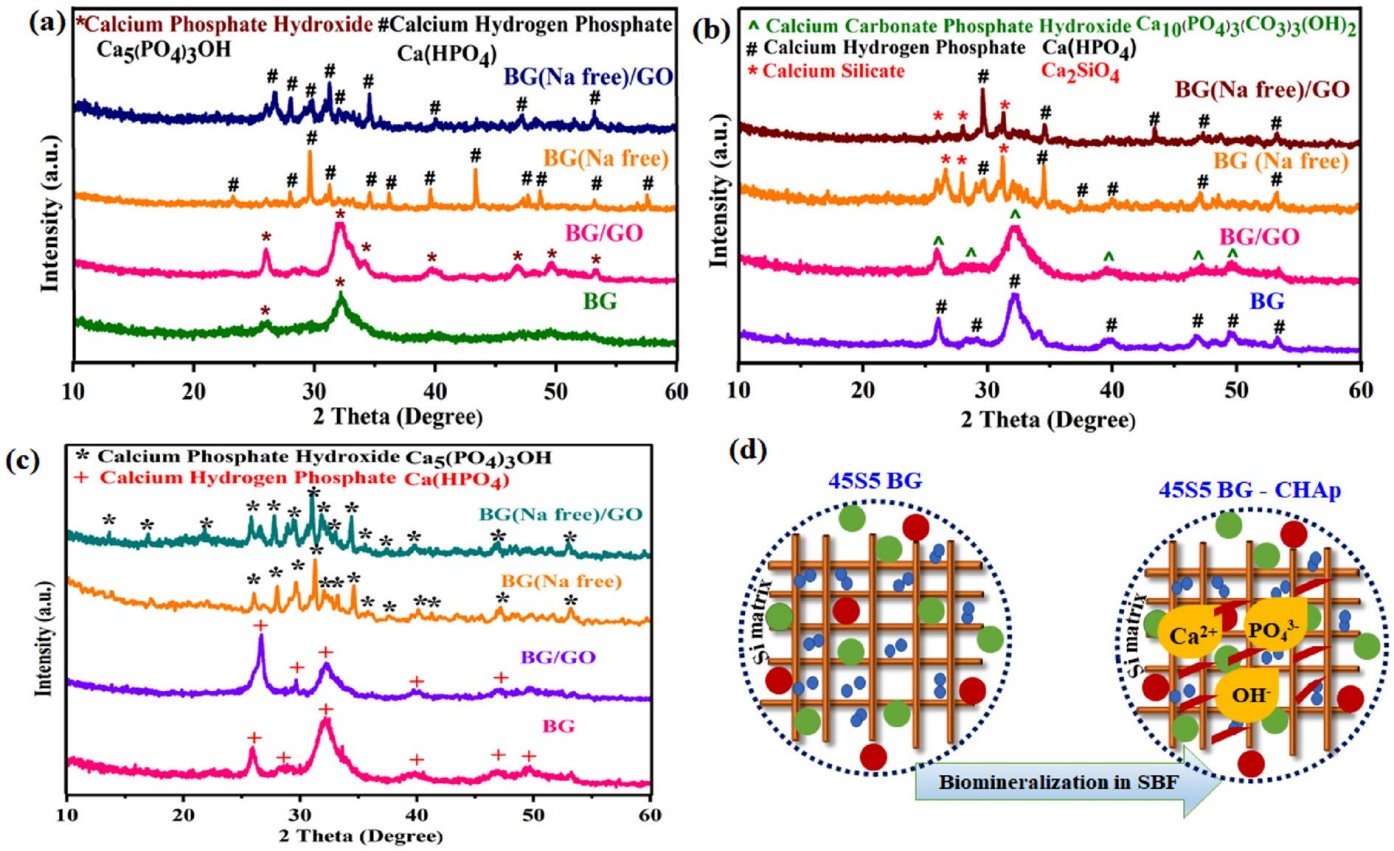
XRD patterns for BG, BG (Na-free), GO and BG/GO, BG (Na-free)/GO nanocomposites after mineralization; (**a**) 1st day, (**b**) 14th day, (**c**) 28th day, and (**d**) illustration of ion-exchanged CHAp formation.

X-ray diffraction (XRD) patterns of mineralized samples are presented in Fig. 1. The formation of hydroxy carbonate apatite (HCA) at 32° and 29° with calcium phosphate hydroxide, calcium hydrogen phosphate, and calcium silicate phases on the 1st and 14th day of mineralization is displayed in Fig. 1a,b. Furthermore, the fully crystallized carbonated hydroxyapatite layer (CHAp) was developed on the 28th day that was confirmed by observing peaks at 2θ values of 26.6°, 31.2°, 32.2°, and 34.2° (Fig. 1c), which correspond to (002), (211), (300), and (202) diffraction planes, as evidenced by enhanced peak intensity and peak shift in the apatite phase (Fig. 1d). With increased immersion duration and amorphous nature, the diffraction peaks of the Na_4_Ca_4_(Si_6_O_18_) phase for BG were decreased^27^.

**Figure 2:**
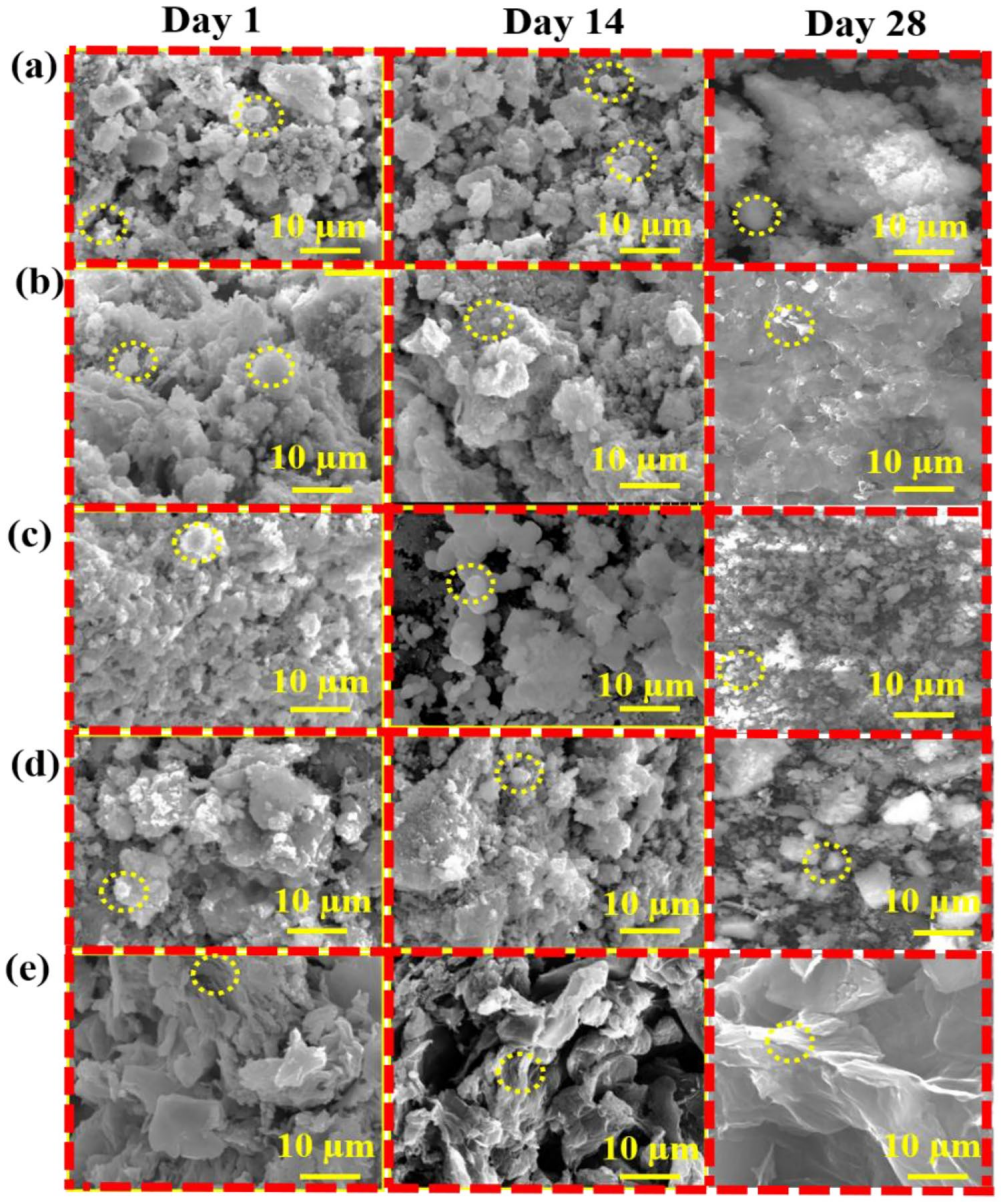
Scanning Electron Microscope (SEM) images of (**a**) BG, (**b**) BG/GO, (**c**) BG (Na-free), (**d**) BG (Na-free)/GO, and (**e**) GO nanocomposites after mineralization.

**Figure 3:**
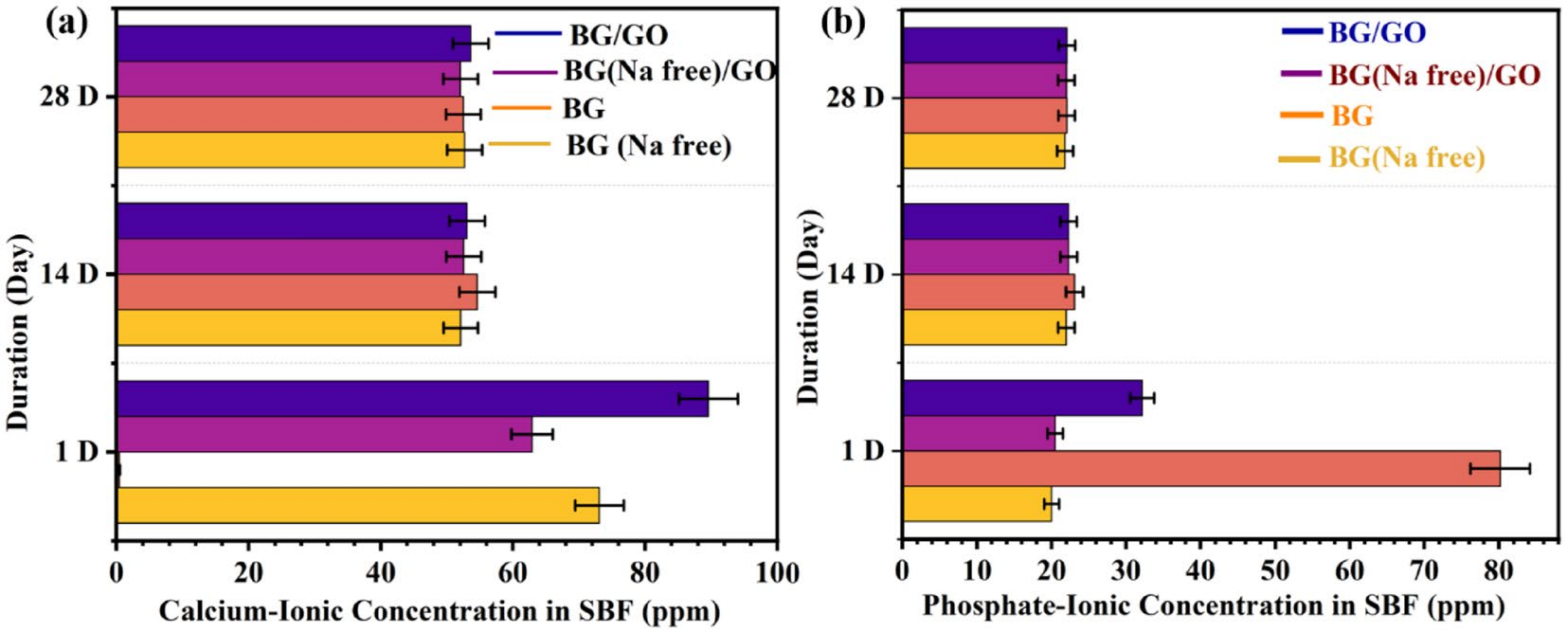
Determination of Ca and P concentration in SBF for different immersion days; (**a**) calcium ion concentration, (**b**) phosphate ion concentration.

The morphology of mineralized samples observed on the 1st, 14th, and 28th days was shown in Fig. 2a–e. The BG and BG (Na-free) exhibited sphere-like morphology as the CHAp layer grew on the surface. BG has a spherical shape with a smooth surface by inhibiting the rod and flake-like morphologies. Compared to the morphology before mineralization, the nanocomposites display rice and ball-like shape with GO sheet separation (Fig. 2a,b). After mineralization, the stacked layers of GO were separated into a single layer by absorbing additional cations from the SBF solution (Fig. 2e). From the BG/GO nanocomposites, the layers for GO sheets with porous character and mineral deposition were visible. Finally, the GO reveals sheet-like shapes as thin layers, confirming the stacked-layer separation using Raman analysis of the 2D area in the GO Raman spectra. After immersion in SBF for various time intervals, the elemental composition of BG, BG (Na-free), GO, BG/GO, and BG (Na-free)/GO nanocomposites were shown in Supplementary Fig. S1. After immersion, the atomic percentages of Ca and P atoms increased, but the atomic percentage of Si atoms decreased^28^. These findings revealed that the BG created an ECM using the CHAp layer in a short period (Fig. 2c,d). The Ca and P concentrations in SBF after immersion was also measured using Inductively coupled plasma–optical emission spectrometry (ICP-OES). Ca and P ion concentrations increased on the 1st day and decreased on the 14th and 28th day, as shown in Fig. 3a,b. Compared to P ions, the Ca ion has a substantially greater concentration on the 1st day, indicating that CHAp rather than hydroxyapatite (Hap) layer development is required for soft tissue regeneration. Na^+^ was detected only in the BG at the same time. These findings are consistent with the energy-dispersive X-ray spectroscopic analysis (EDS) findings.

### Hemoclot assay

**Figure 4:**
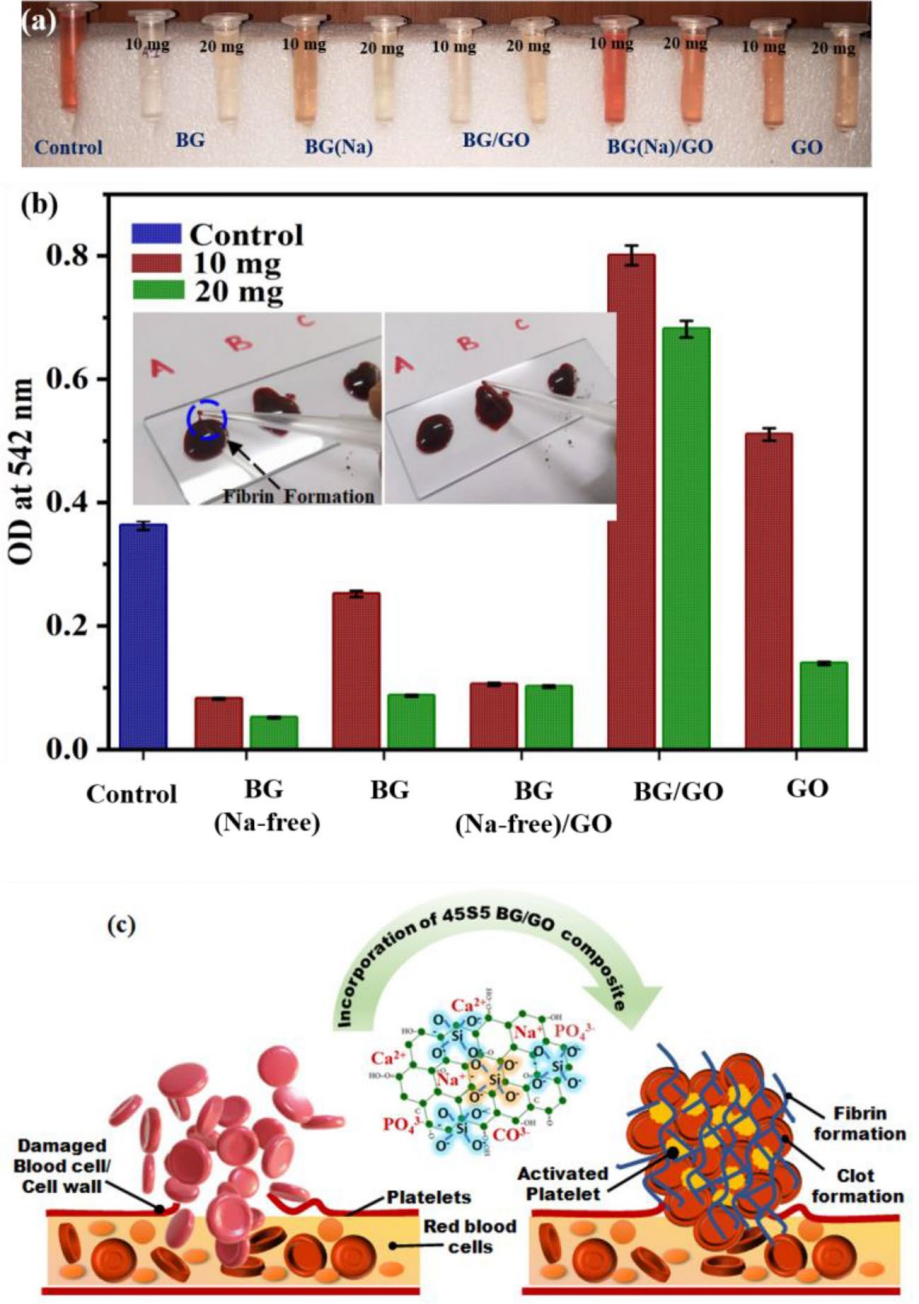
Hemoclot Assay of BG, BG (Na-free), GO and BG/GO, BG (Na-free)/GO nanocomposites; (**a**) photographs of supernatant collected, (**b**) optical density (O.D.) representation of hemoclot for 10 and 20 mg samples, and photographs of a blood clot with fibrin formation (inset), (**c**) schematic representation of hemostatic behavior of 45S5 BG-GO Nanocomposite.

The blood clot study was conducted to determine the currently proposed materials clotting time efficiency using three different methodologies (Thrombogenic, UV Absorption, and Le and White method). Several specific assays are available to determine hemoclot activity as shown in Fig. 4c. Hence, we intend to compare the most studied methods to investigate the material’s performance against effective hemoclot formation^29^. For the UV absorption method, the BG (Na-free) has a 3 min clotting time shown in Fig. 4b, and their corresponding supernatant after UV analysis is displayed in Fig. 4a. Furthermore, the BG has a longer clotting time because it has the potential to break RBCs by increasing osmotic pressure in the fluid due to the Na content^30^. Due to the less hydrophilic character of rGO, the interaction of blood with BG (Na-free)/GO nanocomposites and GO samples consumed a long time due to the unstable rGO presence with less absorption and exposure to a wide surface led to the formation of multiple layers^31^. During composite preparation, GO may be converted to rGO. Further, the thrombogenic method exhibits a shorter clotting time for BG (Na-free) than the UV absorption method. Similarly, the hemoclot activity was studied at room temperature using the thrombogenic activity and Le and White method^32^ were shown in Supplementary Fig. S2a,b, and the clot duration was calculated and presented in Table 1.

**Table 1:** Hemoclot assay of BG, BG (Na-free), GO and BG/GO, BG (Na-free)/GO nanocomposites using UV absorption technique and the Le and White method.

Sample	Thrombogenic activity	UV absorption (mins)	O.D. value (± 0.02) (10 mg)	O.D. value (20 mg)	Le and White’s method
Control	15 min	< 10	0.4783	0.3628	5 min 58 s
BG	6 min 53 s	< 10	0.2517	0.0874	> 10 min
BG/GO	7 min	< 10	0.80085	0.6813	> 10 min
BG (Na-free)	7 min 26 s	< 10	0.08225	0.05205	3 min
BG (Na-free)/GO	6 min	< 10	0.1064	0.10235	4 min 12 s
GO	5 min	< 10	0.5103	0.13935	4 min 2 s

Ca ions in 45S5 BG play a vital role in coagulation by stimulating the development of clotting factors (IV) thrombin and fibrin^33^. Because of the release of Na with increasing alkaline pH and osmotic pressure, the BG has a longer clotting time. As a result, the RBCs may be lysed by Na^34^. Due to the mild hydrophobic character of rGO, the BG/GO and BG (Na-free)/GO nanocomposites had a longer clotting time. Owing to the transition of GO to rGO reduction and the porous nature of BG, the contact angle measurement establishes the hydrophilic nature of BG (Na-free) and slight hydrophobicity of BG/GO and BG (Na-free)/GO nanocomposites Supplementary Fig. S3. The hydrophobic characteristic of composites prevents lipid layers of RBCs from interacting with the GO surface, potentially lowering blood clotting capacity and lengthening clotting time. As a result, the hemostatic data is supported by the contact angle measurements.

### Hemocompatibility assay

**Figure 5:**
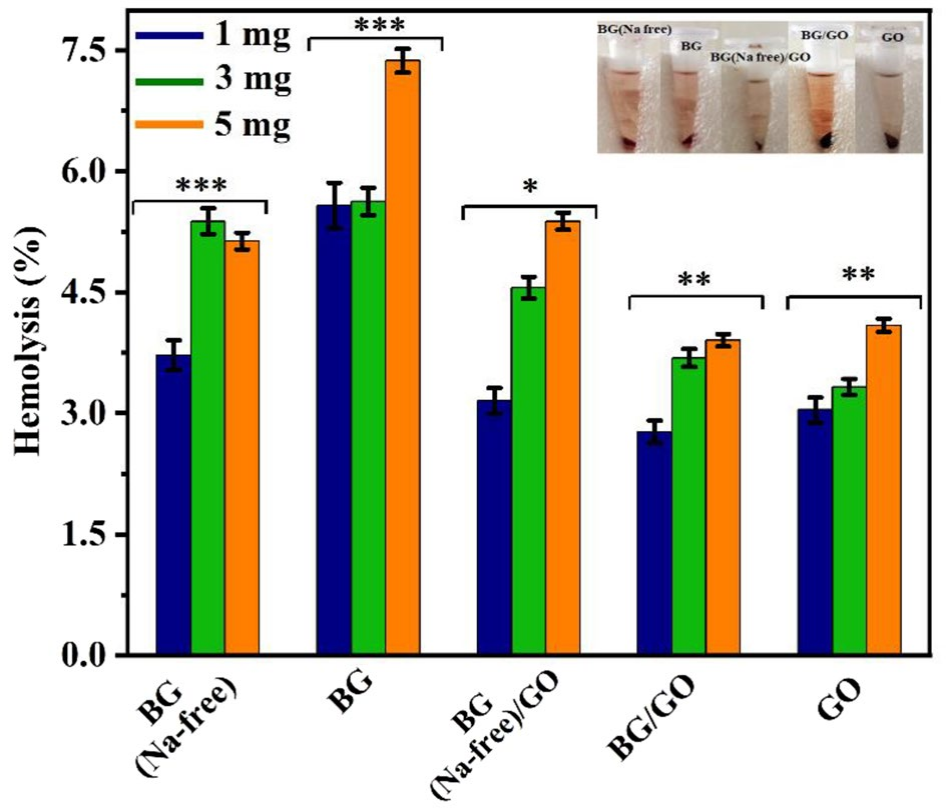
Hemolysis assay for BG, BG (Na-free), GO and BG/GO, BG (Na-free)/GO nanocomposites for varying concentrations and inserted photographs of haemolysed samples. The asterisks indicate significant difference (*p < 0.09, **p < 0.02, ***p < 0.0318).

Hemocompatibility of the fabricated nanocomposites was evaluated using human blood to assay the process of hemolysis. According to International Organization for Standardization (ISO) 10993-4 standards, wound healing products with a hemolytic value of less than 5% are considered safe^35^. The hemolysis assay was used to measure the rupture rate of RBCs in BG, BG (Na-free), GO and BG/GO, BG (Na-free)/GO nanocomposites with three distinct concentrations of 1, 3, and 5 mg, with weighted samples duplicated as 950 µL PBS + 50 µL RBCs. According to the findings, BG (Na-free) had the least amount of lysis (Fig. 5). However, the presence of Na in BG enhanced erythrocyte lysis^36^. GO with BG (Na-free) composites exhibited good compatibility for 1 mg, but GO with BG composites showed mild lysis at 3 and 5 mg. Compared to the 1 and 3 mg samples, the 5 mg samples have a higher lysis rate. According to ASTM standards (ASTM F 756), all of the fabricated composites had very little lysis and are thus considered an excellent hemocompatible material to take forward.

### Antibacterial activity studies

**Figure 6:**
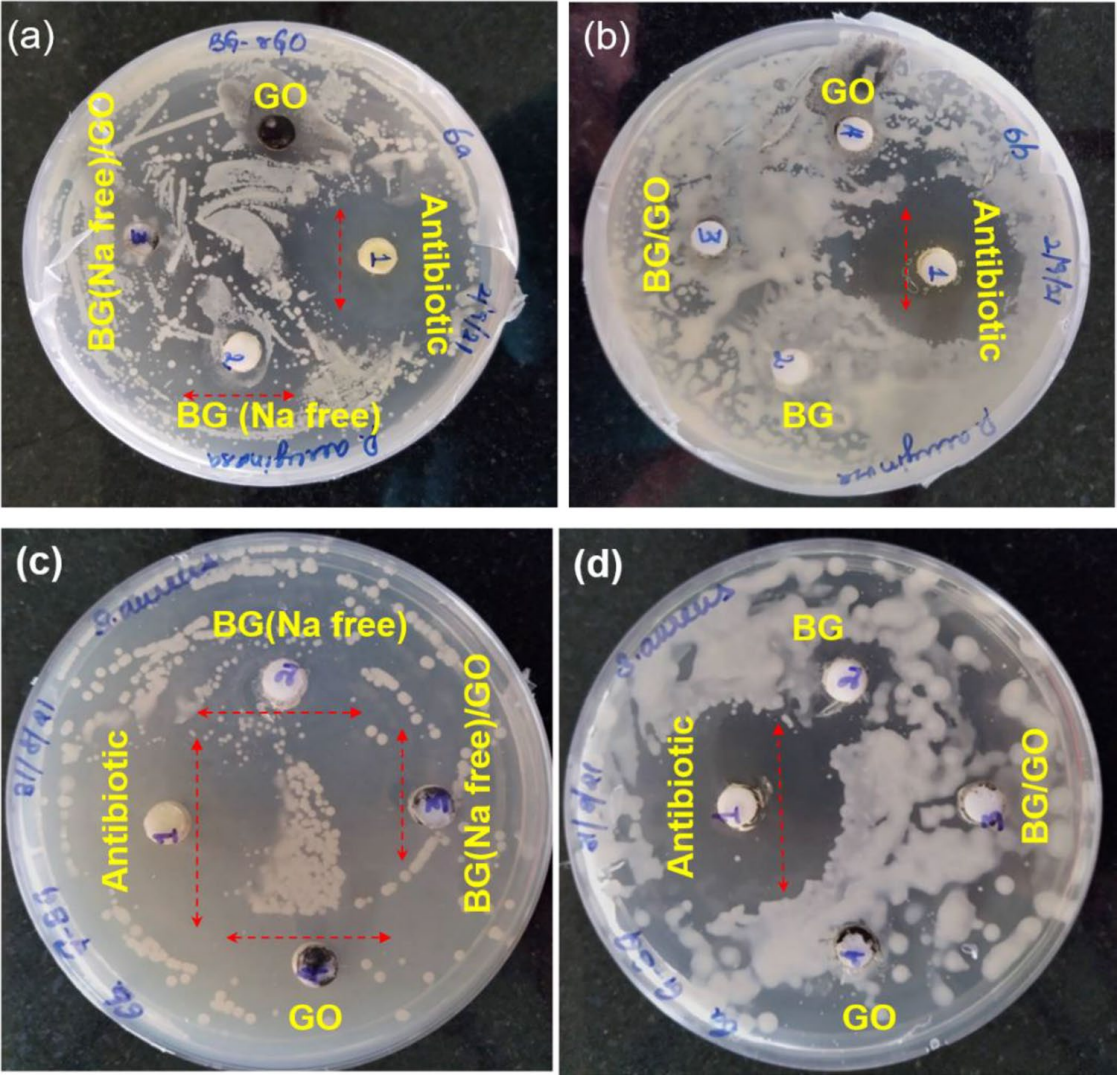
Photographs of antibacterial activity with (**a**) BG (Na-free), GO and BG (Na-free)/GO nanocomposite against *P. aeruginosa*, (**b**) BG and BG/GO nanocomposite against *P. aeruginosa*, (**c**) BG (Na-free), GO, and BG (Na-free)/GO nanocomposite against *S. aureus*, (**d**) BG and BG/GO nanocomposite against *S. aureus.*

BG, BG (Na-free), GO and BG/GO, and BG (Na-free)/GO nanocomposites were tested for antibacterial activity against *P. aeruginosa* and *S. aureus*. According to the findings, BG, BG (Na-free), GO, BG/GO, and BG (Na-free)/GO all had an excellent antibacterial impact against gram-positive and gram-negative bacteria. Furthermore, compared to antibiotics (Tetracycline hydrochloride), BG and BG/GO had modest antibacterial efficacy against *S. aureus*, a gram-positive bacterium in Fig. 6a–d. The increased release of alkali ions Na in the medium bacterial harm the bacterial cell membrane^37^. Because of their pointed rod-like form, the BG (Na-free) particles have a better antibacterial impact, damaging the bacterial membrane’s cell wall and causing bacterial death^38^ as it is known as the one-dimensional material with definite high index phase setting as more surface energy. Several defects and oxygen vacancies exist in the GO, which might interact with water molecules to form reactive oxygen species (ROS)^39^. By preventing the transition of a bacterial membrane, these ROS will trigger bacterial death^40^. As a result, increased structural instability in GO and rGO leads to increased bacterial mortality and anti-bacterial action.

**Figure 7:**
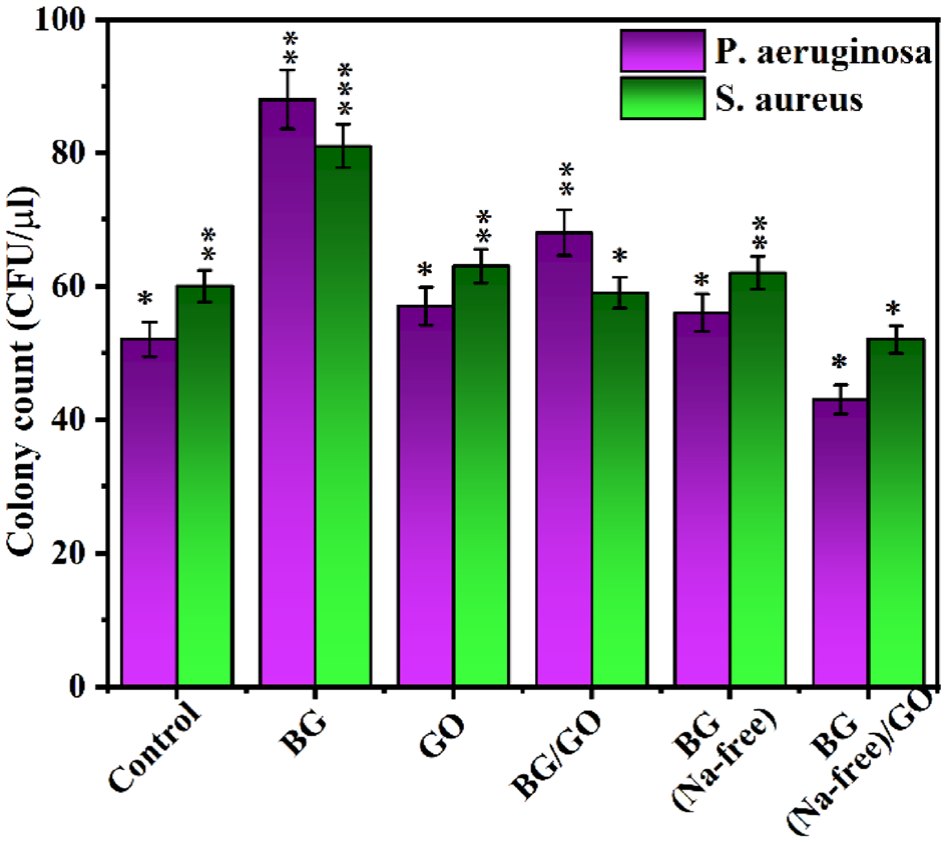
Antibacterial activity of BG, BG (Na-free), GO, BG/GO, and BG (Na-free)/GO nanocomposites against *S. aureus* and *P. aeruginosa* bacteria. The asterisks indicate significant difference *p < 0.02, **p < 0.03, ***p < 0.05.

BG, BG (Na-free), GO, BG/GO and BG (Na-free)/GO nanocomposite revealed adequate antibacterial activity against the *S. aureus* and *P. aeruginosa* bacteria (Fig. 7). GO, and BG (Na-free) shows an equal level of response when compared to control. BG (Na-free)/GO nanocomposite shows less viability of bacteria than the control. Also, the antibacterial activity was decreased with the addition of GO for *S. aureus*, and there is no significant observable difference between *S. aureus* and *P. aeruginosa* bacteria*.* Moreover, the BG and BG/GO samples with increased bacterial formation are due to the hydrophobic nature of BG/GO and generally, the BG’s Na ions may leach out easily from the BG matrix and cause damage to the bacterial cell membranes which leads less bacterial resistance. Overall, the results confirmed that the incorporation of GO into the BG matrix is a notable approach to improving antibacterial activity.

### Cell viability and cell proliferation assay

**Figure 8:**
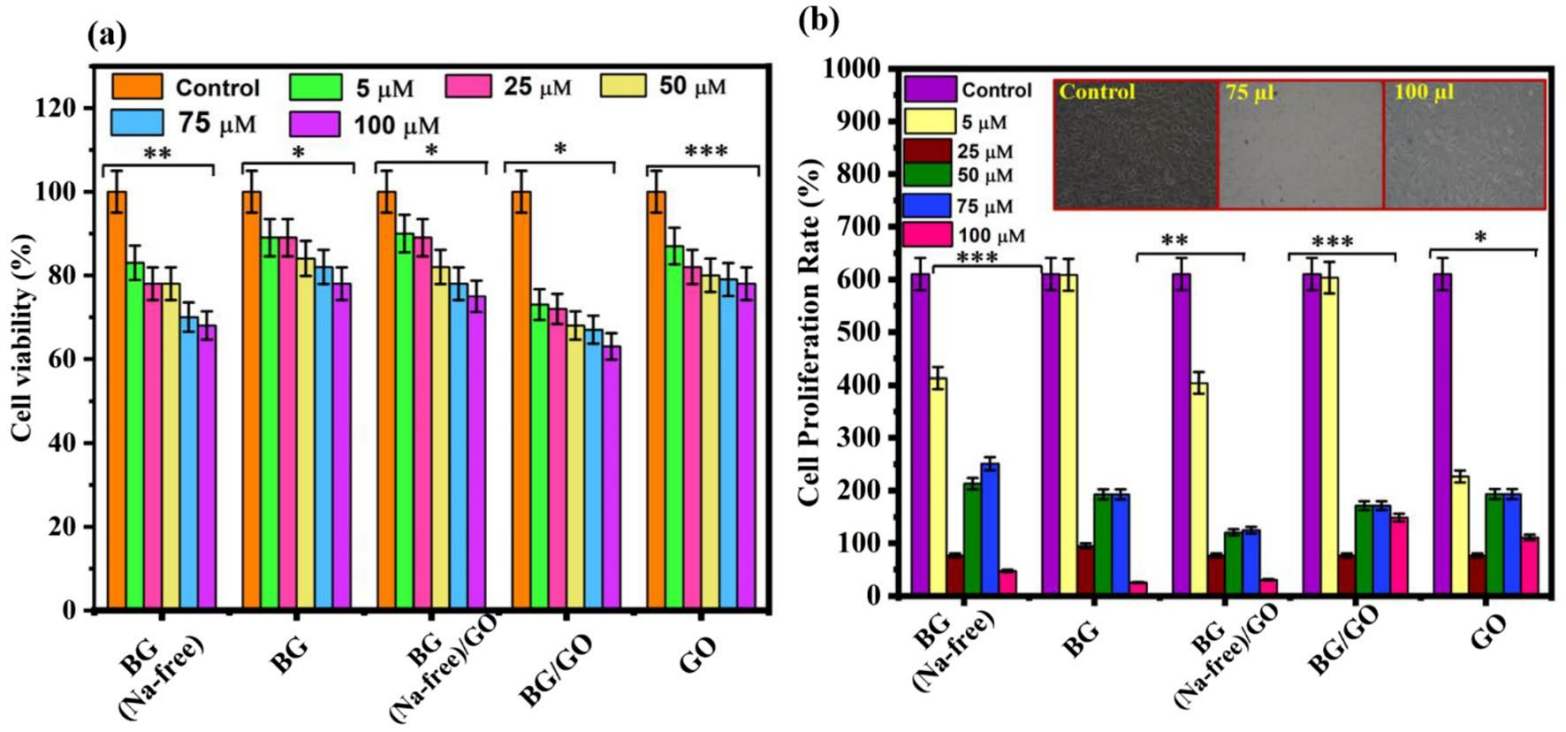
Cytocompatibility and cell proliferation properties; (**a**) cell viability of BG, BG (Na-free), GO and BG/GO, BG (Na-free)/GO nanocomposites over L929 for the incubation for 24 h. The asterisks indicate significant difference (*p < 0.02, **p < 0.08, ***p < 0.02), (**b**) cell proliferation of fibroblast cells on BG, BG (Na-free), GO and BG/GO, BG (Na-free)/GO nanocomposites for 72 h incubation. The asterisks indicate significant difference (*p < 0.015, **p < 0.045, ***p < 0.068).

The cytotoxicity of 45S5 BG and GO must be assessed for wound healing applications. Since the BG, BG (Na-free), GO, and BG/GO, BG (Na-free)/GO nanocomposites directly interacted with L929 cells as a direct technique. L929 cells are normal immortalized cell lines from the mouse’s subcutaneous connective tissue, which was used to determine dose-dependent cytotoxicity after 24 h of incubation. Furthermore, roughly 85% of fibroblast cells were metabolically active at the higher concentration of samples (100 μM), indicating a modest cytotoxic response (Fig. 8a). The proliferation stage of fibroblast cells was driven by several growth factors like VEGF, transforming growth factor (TGF), and matrix metalloproteinases (MMPs) for angiogenesis formation. For BG (Na-free) particles, the cells had a spindle-shaped morphology and excellent cell formation^41^. Furthermore, other samples do not exhibit a noticeable rate of proliferation, as the GO has a lower rate of proliferation, while the BG/GO and BG (Na-free)/GO nanocomposites have a higher rate of proliferation, as the BG (Na-free) causes cell growth via fibroblast growth factors (Fig. 8b). At 72 h, the proliferation rate was increased for BG (Na-free) and gradually increased for BG/GO and BG (Na-free)/GO nanocomposites.

### Apoptosis assay

**Figure 9:**
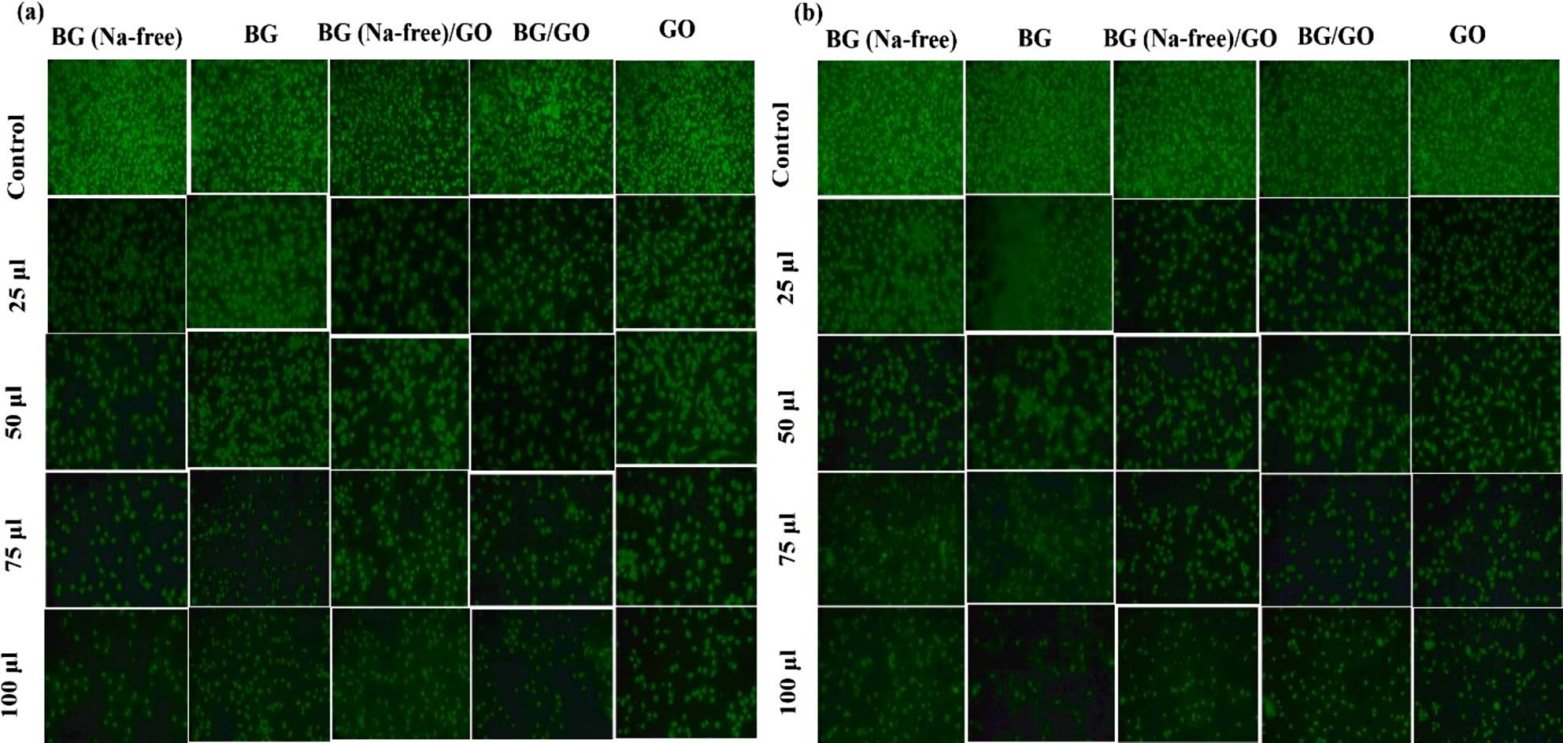
Apoptosis assay of L929 cell treating with the following samples; (**a**) upto 24 h and (**b**) upto 48 h.

The apoptotic test was used to further clarify the in vitro cytocompatibility of BG, BG (Na-free), GO, and BG/GO, BG (Na-free)/GO nanocomposites. The current study used the differential uptake of acridine orange and ethidium bromide by L929 cells to detect apoptosis. The interpolating fluorescent stain, cell-permeable acridine orange, generates consistent green fluorescence from both living and non-viable cells. The apoptotic cells were brightly labeled with the usual chromatin condensation and nuclear fragmentation development^42^. After 24 and 48 h of incubation, the excellent confluence of live cells with bright-field green fluorescence was seen when BG and BG (Na-free) interacted with cell DNA. The cell counts steadily declined when the concentration was increased from 25 to 100 μL, and an ideal range of cell density and morphology was found at 50 μL for all samples in Fig. 9a,b. Break-in ribonucleic acid in the monolayer might cause a change in cell morphology. When compared to control, both 45S5 BG and GO samples retain their cell structure up to 48 h were seen in Fig. 9. Compared to 24 h, cell density was low at 48 h at a concentration of 100 μL. These findings are consistent with cell viability and proliferation data. The density of live cells acridine orange (AO) stained increased at 50 and 75 μL concentrations; however, at high concentrations (100 μL), BG and GO showed slightly less density of viable cells than BG (Na-free), BG/GO, and BG (Na-free)/GO nanocomposites. At the end of the 48-h apoptosis experiment, greater cell density (increase in AO living cells) was detected.

### Scratch assay

**Figure 10:**
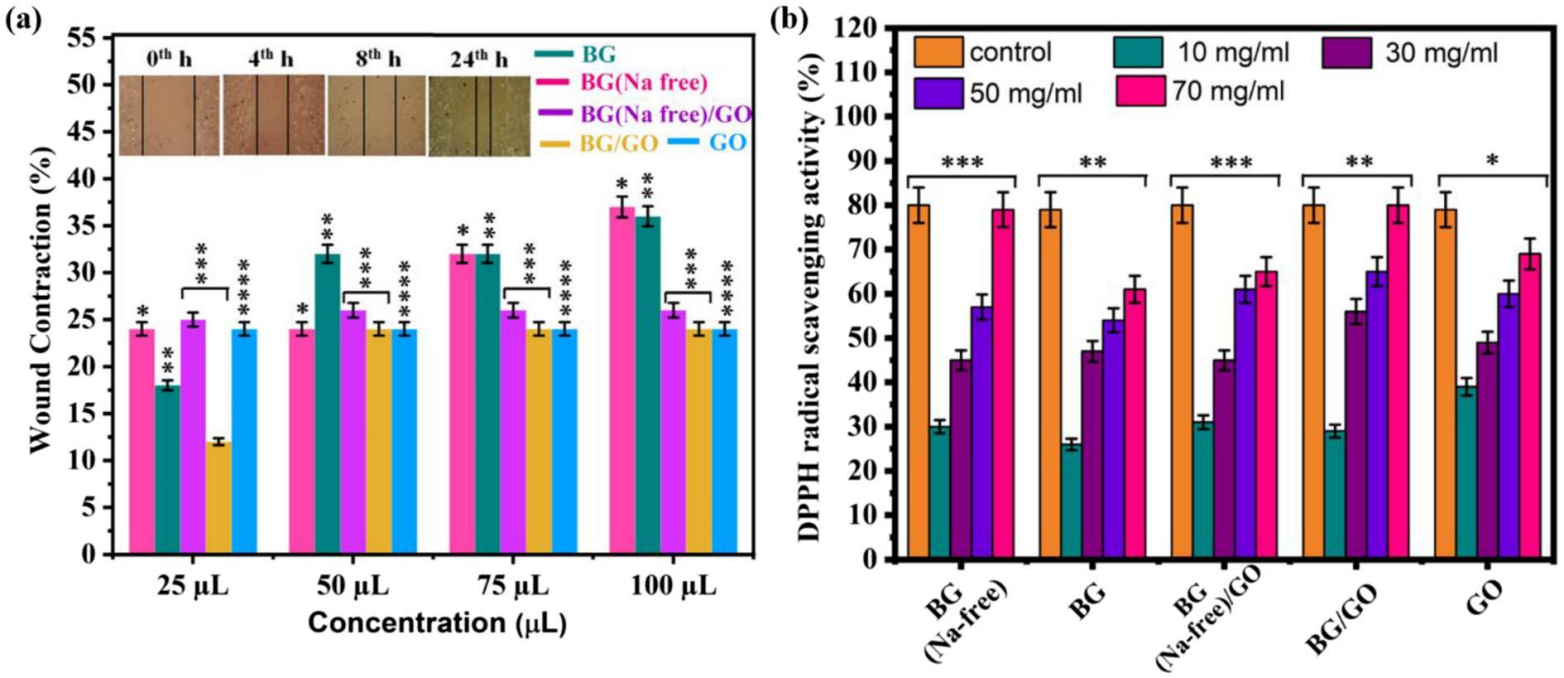
(**a**) Scratch assay of L929 fibroblast cells treated with BG, BG (Na-free), GO, and BG/GO, BG (Na-free)/GO nanocomposites. The asterisks indicate significant difference (*p < 0.05, **p < 0.017, ***p < 0.022, ****p < 0.055), (**b**) antioxidant activity of BG (Na-free), BG, GO, and BG/GO, BG (Na-free)/GO nanocomposites was measured using the DPPH radical scavenging assay. The asterisks indicate significant difference (*p < 0.0003, **p < 0.023, ***p < 0.047).

BG, BG (Na-free), GO, and BG/GO, BG (Na-free)/GO nanocomposites demonstrated moderate wound contraction with varying concentrations up to 24 h, as illustrated in Fig. 10a. Compared to nanocomposites, BG (Na-free) and BG at higher concentrations (100 μL) have potent healing properties. The GO, BG/GO, and BG (Na-free)/GO exhibit only 25% of wound closure due to less interaction of rGO with cell membranes. These results are in good concurrence with cell proliferation assays. However, compared to the control, all samples have reduced wound contraction. The primary reason for reduced contraction of cells with BG, BG (Na-free), GO, and BG/GO, BG (Na-free)/GO nanocomposites is the lower concentration (0.1 mg/mL) which is tenfold lower than typical for 24 h incubation duration. The low concentration slowed cell movement. It is also implied that extending the healing time beyond 36 h might result in full-area cell migration in the samples. However, there is a sustainable formation (40%) in the cell line for BG and BG (Na-free) samples at the concentration of 50, 75, and 100 μL.

### DPPH assay

In the intricate symphony of diabetic wound healing, multiple elements such as hyperglycemia, neuropathy, blood supply, matrix turnover, wound contraction, and the microbiome plays a vital role^43^. It is now recognized that oxidative stress reduction is vital in diabetic wound healing. Overproduction of ROS is caused by an imbalance of free radicals and antioxidants in the body, which leads to cell, tissue, and wound damage^44^. As a result, lowering ROS levels through antioxidative systems may help to enhance healing by reducing oxidative stress-induced damage^45^. On the other hand, excessive generation of defective ROS detoxification causes rapid inflammation and oxidative stress-induced cellular damage, which is the primary cause of wound healing delays^46^. For dose-dependent relationship assay (10, 30, 50, 70 mg), the BG (Na-free), BG, GO, and BG (Na-free)/GO, BG/GO nanocomposites, the scavenged DPPH radicals show enhanced activity in Fig. 10b shows the DPPH activity for BG (Na-free), BG, GO, BG (Na-free)/GO, and BG/GO nanocomposites simultaneously depends upon concentration. The DPPH activity values are comparatively enhancing for 10, 15, and 20% of BG/GO, etc. When the concentration of BG (Na-free), BG, GO, BG (Na-free)/GO, and BG/GO nanocomposites is gradually increased, the scavenging activity increases. Furthermore, the BG (Na-free) and BG/GO composite had the highest scavenging effect. The radical formation in graphene-based materials is caused by sp^2^ carbons and their delocalized spin across the graphene sheets, which is considered a significant factor in the destruction of radical generation via electron transfer^47^. The free radical scavenging activity of the 45S5 BG and GO against the production of hydroxyl radicals and the oxidation process was confirmed by the DPPH assay.

### Raman spectra analysis

**Figure 11:**
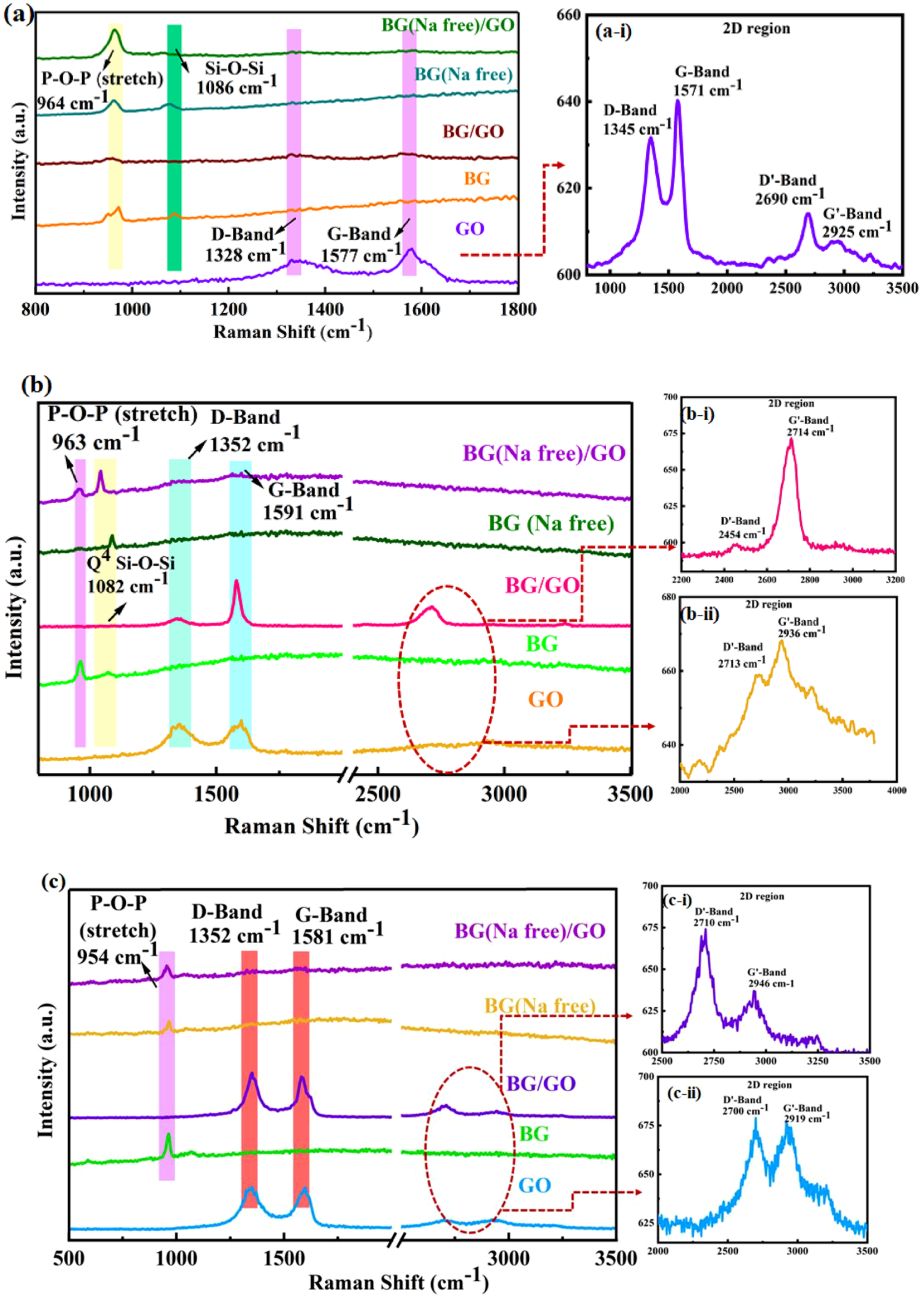
Raman spectra of BG, BG (Na-free), GO and BG/GO, BG (Na-free)/GO nanocomposites after mineralization; (**a**) 1st day; (**a-i**) 2D-region of GO, (**b**) 14th day; (**b-i,b-ii**) 2D-region of BG/GO and GO, (**c**) 28th day; (**c-i,c-ii**) 2D-region of BG/GO and GO.

**Figure 12:**
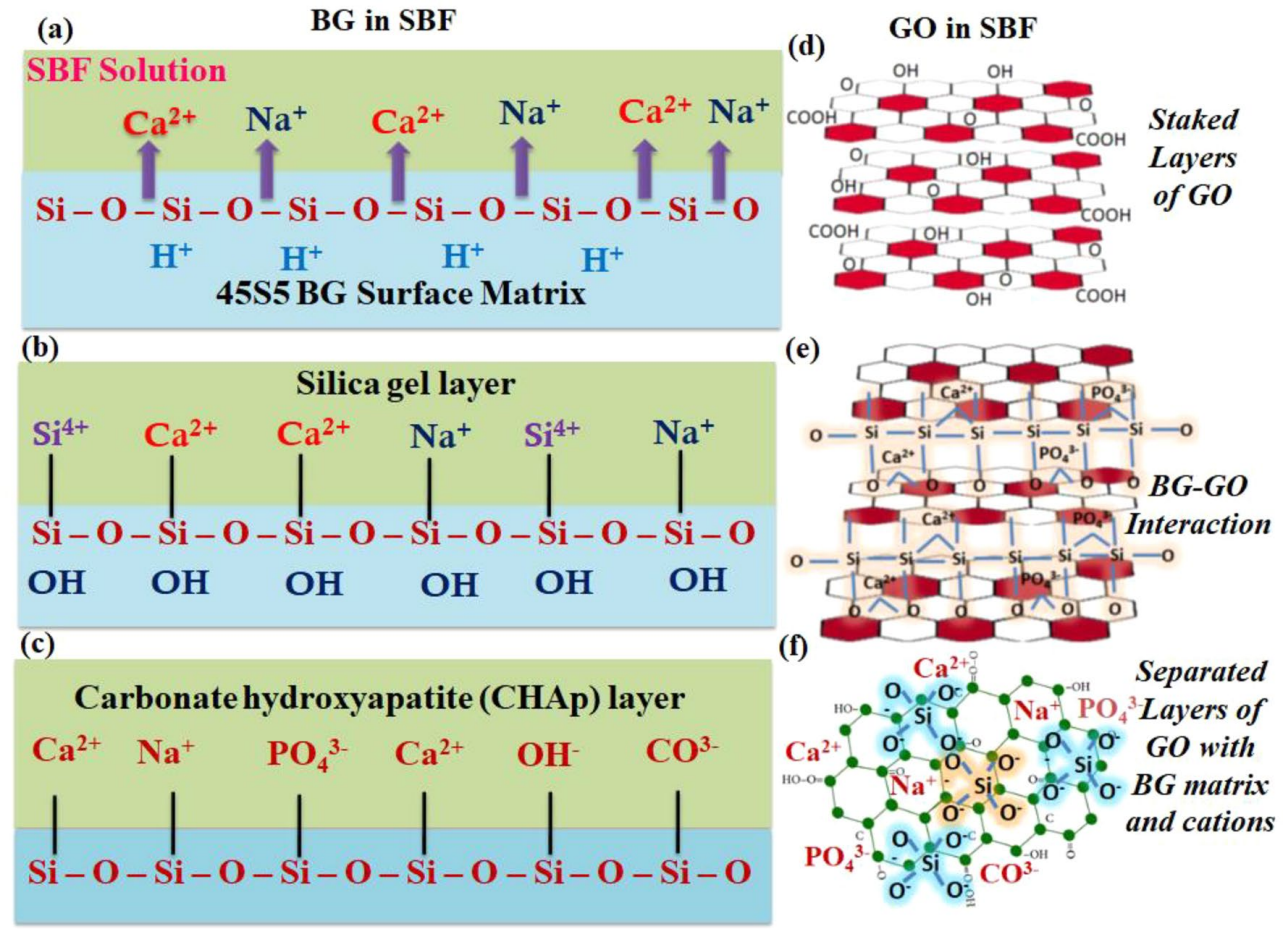
Schematic representation of structural modifications of BG and GO immersed in SBF solution; (**a**) BG immersed in SBF with cationic ion exchange, (**b**) formation of Si layer by dissolving Ca and P ions, (**c**) CHAp layer formation on the BG surface, (**d**) stacked layers of GO in SBF solution, (**e**) GO sheets interact with BG matrix and cations in SBF via hydroxy and carbonyl groups, which reduces the interplanar distance and weakens the van der Waals force between the sheets, (**f**) separated GO stack into a single layer with BG matric interconnection.

The Raman spectra for mineralized samples were obtained on the 1st, 14th, and 28th days and are displayed in Fig. 11 Furthermore, the bonds with I_D_/I_G_ values are listed in Supplementary Table S1. The phosphate (P–O–P) stretching peak was seen at 964 cm^−1^ (1st day), 963 cm^−1^ (14th day), and 954 cm ^− 1^ (28th day), indicating the formation of apatite layers via phosphate stretching mode in addition to Si–O–Si stretching mode. The apatite layer development was well initiated on the 1st day and confirmed on the 28th day. Furthermore, the GO-incorporated BG exhibits a more remarkable shift in the phosphate bond and the D and G bands. The presence of functional groups in the GO influences the mineralization process. After mineralization, the 2D region formed for GO shows fluctuations in the number of graphene layers^48^. GO functional groups (–COOH, –OH) interact with the inorganic salts (Na^+^, Ca^+^, P^+^) in the SBF solution during the mineralization process and undergo charge transfer, which tends to exfoliate the GO layer as shown in Fig. 12a–f. It is anticipated that the exfoliation of the GO sheet may interrupt the GO’s interplanar distance due to cation intercalation and leading to weakening of the Vander Waals force^49^; which facilitates GO sheet separation which affects the 2D band, as shown in Fig. 11a-i,b-ii,c-ii Because of the presence of Na^+^ in the BG (Na-free), the BG/GO composite alone displays 2D bands in Fig. 11b-i,c-i. The amount of Na^+^ in the nanocomposites affects the exfoliation of GO layers during immersion. The non-bridging oxygen in 45S5 BG formed an electrostatic interaction with the GO functional groups. The carboxyl group and oxygen vacancies in GO attract positively charged cations when they are in contact with the SBF solution, and the ions are deposited in the GO, increasing the interlayer spacing and forming separate sheets.

### Material characterization

#### Structural analysis

**Figure 13:**
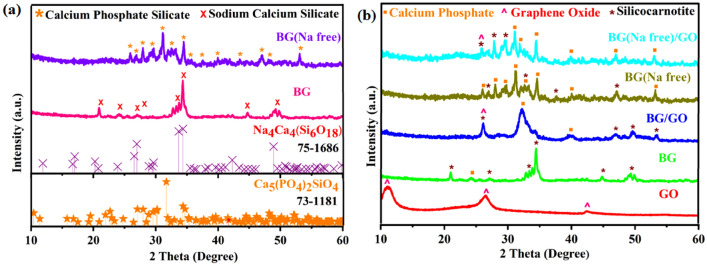
XRD patterns for (**a**) BG and BG (Na-free), (**b**) BG, BG (Na-free), GO and BG/GO, and BG (Na-free)/GO nanocomposites.

XRD patterns were indexed and identified the orientation of the prepared 45S5 BG crystal structure shown in Fig. 13a. BG has a sodium calcium silicate (Na_4_Ca_4_(Si_6_O_18_)) phase with a combeite structure^50^ (ICDD card no. 75-1686), whereas BG has a calcium phosphate silicate (Ca_5_(PO_4_)SiO_4_) phase with silicocarnotite crystal structure (ICDD card no. 73-1181). Furthermore, due to the development of the BG-GO matrix, the XRD patterns of the BG/GO and BG (Na-free)/GO nanocomposites exhibit changed crystal orientation. The GO (001), (002), and (100) planes appeared at the 2θ values of 11.09°, 26.47°, and 42.° respectively. Furthermore, due to the inclusion of oxygen functional groups from GO, the BG/GO nanocomposite has a higher intensity than BG were displayed in Fig. 13b. The reduced graphene oxide (rGO) signal produced at 42° confirmed an expansive and amorphous character. Compared to BG (Na-free), the nanocomposite BG (Na-free)/GO exhibits a two-fold increase in intensity and a peak shift. Similarly, the BG/GO nanocomposite exhibits peak widening and shifts a twofold reduction in intensity. The calcium phosphate silicate and calcium phosphate phase are seen in the nanocomposites of BG/GO, and BG (Na-free)/GO, respectively.

#### Morphological analysis

**Figure 14:**
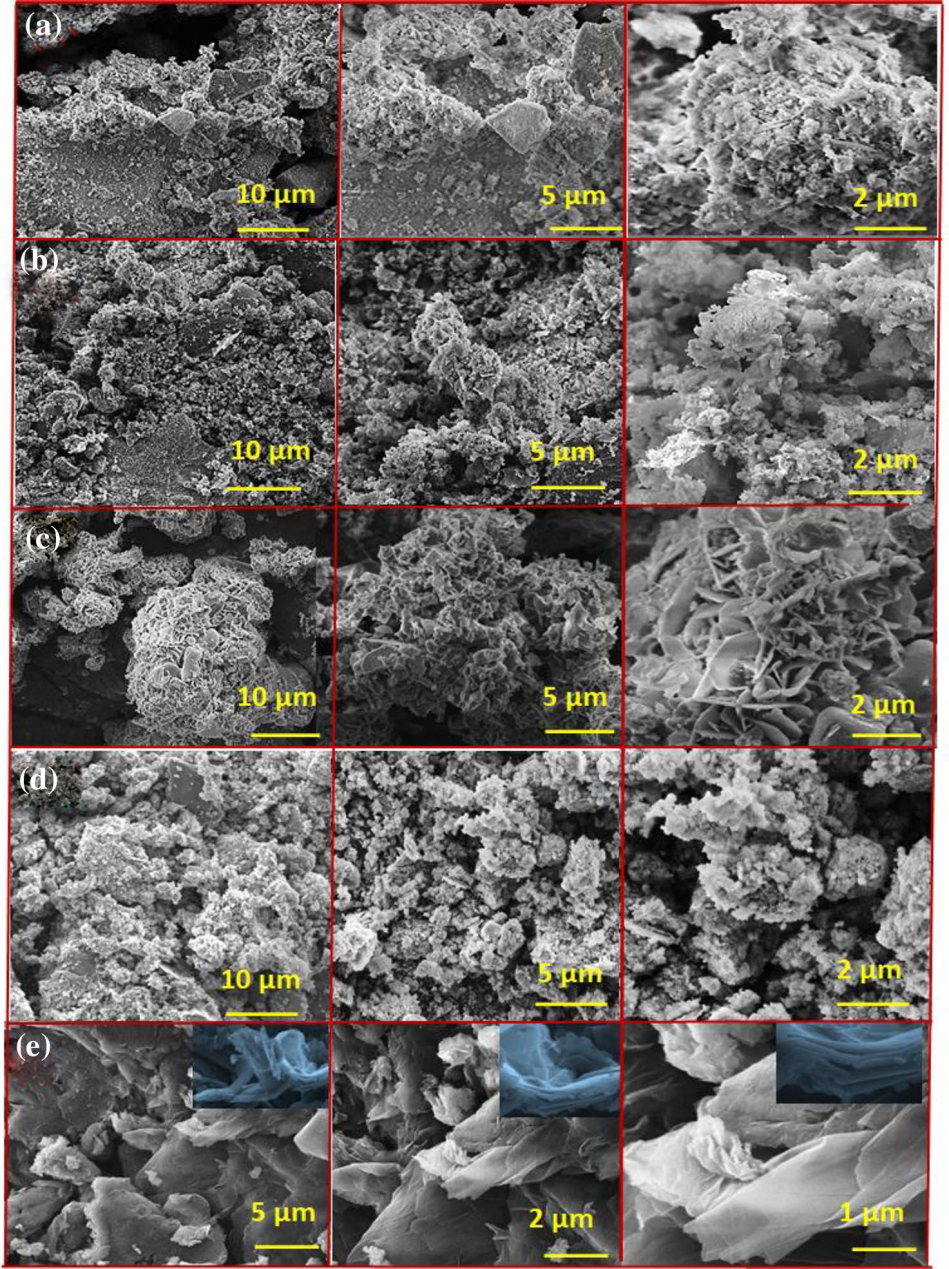
SEM images of (**a**) BG, (**b**) BG/GO, (**c**) BG (Na-free), (**d**) BG (Na-free)/GO, and (**e**) GO.

SEM imaging on the surface of the fabricated nanocomposites was performed to examine the morphology, microstructure, and element distribution displayed in Fig. 14a–e. BG morphology revealed micron-sized spherical particles with cluster formation (Fig. 14a), and BG spheres were decorated over the GO sheets in BG/GO nanocomposites. The fabricated BG (Na-free) has rods and flake-like morphology (Fig. 14c), but when GO was added, the BG (Na-free)/GO nanocomposite had a spherical morphology connected to the GO sheets. The separate GO sheets were observed and shown in Fig. 14e, which depicts 2D stacked and lined layers of sheets. The interaction of functional groups (–COO, –OH) with the non-bridging oxygen sites resulted in the attachment of BG and BG (Na-free) particles on the GO surface in composite samples (Fig. 14b,d). The mapping pictures showed the distribution of the chemical composition of 45S5 BG and GO in Supplementary Fig. S4a–g. In EDS mapping corresponding to the regions covered by GO in nanocomposites, significant signals for Si, Ca, P, Na, and O were observed and indicated the presence and uniform distribution of Si, Ca, and P on the GO surface. High carbon and oxygen signals were also observed for GO sheets. The composition of elements was analyzed using EDA, and their spectrum of BG, BG (Na-free), GO, and BG/GO, BG (Na-free)/GO nanocomposites validated the presence of all necessary components and is exhibited in Supplementary Fig. S5 and weight percentage were tabulated in Supplementary Table S2.

**Figure 15:**
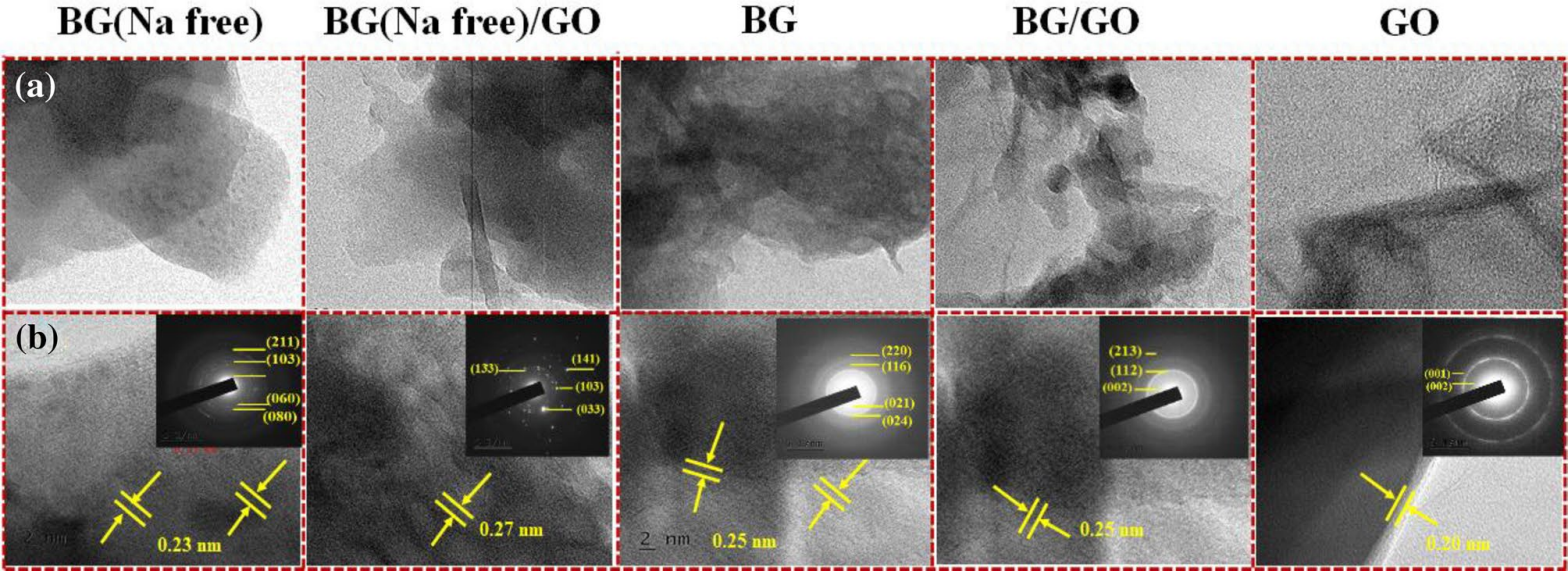
(**a**) HRTEM images of the BG, BG (Na-free), GO, and BG/Na, BG (Na-free)/GO nanocomposites (scale bar: 200 nm), (**b**) corresponding cross-sectional profile (scale bar: 2 nm) at different magnifications, with respective SAED pattern (inset). The arrows show sheet separation.

The BG and BG (Na-free) particles were formed as solid spheres fused with matrix-like surfaces, as exhibited in high-resolution transmission electron microscopy (HRTEM) micrographs of BG, BG (Na-free), GO, and BG/GO, BG (Na-free)/GO nanocomposites (Fig. 15a). The 45S5 BG particles in the nanocomposites are connected to the GO surface in various morphologies, including long rods and microscopic spheres. The morphology of the GO samples resembles a single stacked sheet. With the appropriate planes, the selected area electron diffraction (SAED) pattern determines the amorphous nature of 45S5 BG microcrystals (Fig. 15b inset). Thin black rings in the SAED pattern imply the GO particle formation, while the bright spot indicates 45S5 BG particle formation^51^. The HRTEM data supports the successful formation of the (Na_4_Ca_4_(Si_6_O_18_)) and Ca_5_(PO_4_) SiO_4_) phases as well as the (002) plane for GO, as revealed by the XRD patterns.

#### Compositional analysis

**Figure 16:**
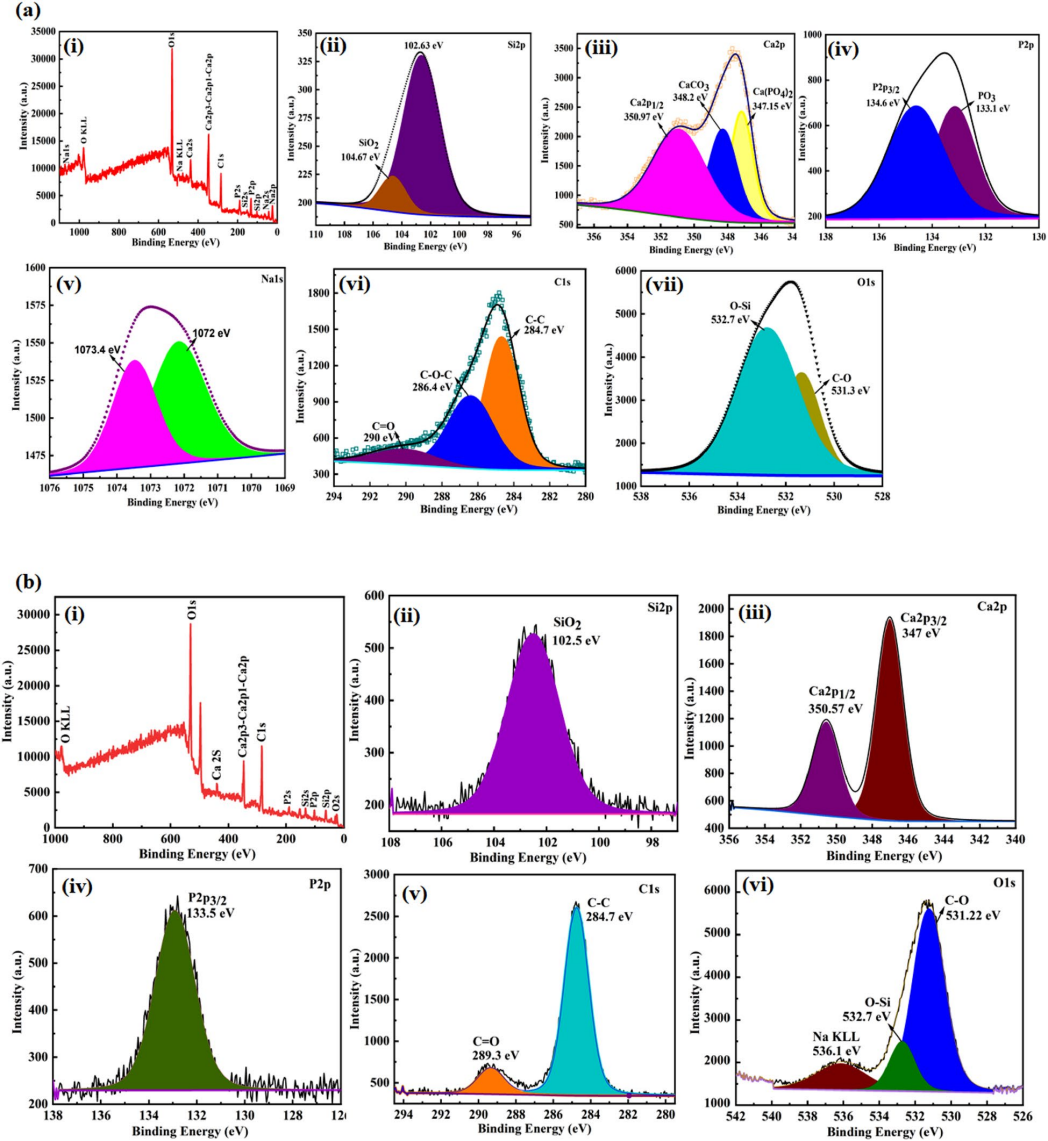

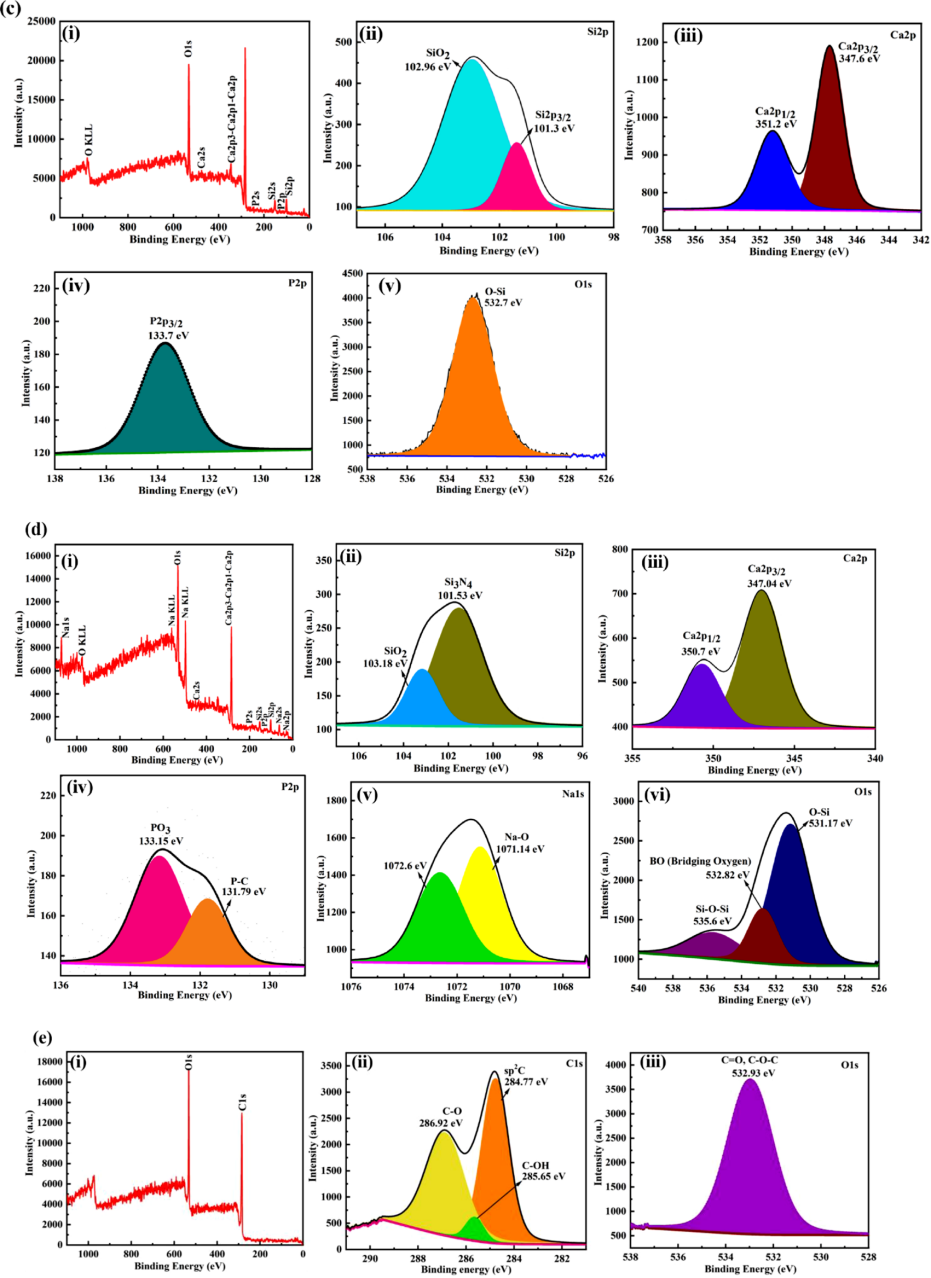
XPS spectra of (**a**) BG/GO, (**b**) BG (Na-free)/GO nanocomposites, (**c**) BG (Na-free), (**d**) BG, (**e**) GO. The survey and core-level spectrum of Si 2p, Ca 2p, P 2p, Na 1s, C1s, and O1s.

The chemical state and composition of BG, BG (Na-free), GO and BG/Na, BG (Na-free)/GO nanocomposites were determined using X-ray photoelectron spectroscopy (XPS), and the spectrum results are given in Supplementary Table S3. The XPS survey spectra determine the relative amounts of silica, phosphorus, calcium, carbon, oxygen, and sodium present in the BG/GO, BG (Na-free)/GO nanocomposites. The survey spectrum shows the presence of Si 2s, Si 2p, P 2s, P 2p, Ca 2s, C 1s, O 2s, O 1s, and O KLL. Further, the survey spectrum of BG/GO shows the presence of Si 2s at 152.23 eV, Si 2p at 99.56 eV, P 2s at 188.60 eV, P 2p at 131.66 eV, Ca 2s at 438.39 eV, Ca 2p3–Ca 2p1–Ca 2p at 346.35 eV, C 1s at 283.65 eV, O 1s at 531.69 eV, O KLL at 979.86 eV, Na 2s at 42.63 eV and Na 1s at 1071.91 eV (Fig. 16ai). The survey spectrum of BG (Na-free)/GO shows the presence of Si 2s, Si 2p, P 2s, P2p, Ca 1s, Ca 2s, Ca2p3–Ca2p1–Ca2p, O 1s and O KLL as shown in Fig. 16bi. The survey spectrum of BG (Na-free) shows the presence of Si 2s, Si 2p, P 2s, P2p, Ca 2s, O 1s and O KLL as shown in Fig. 16ci. The survey spectrum of BG shows the presence of Si 2s, Si 2p, P 2s, P2p, Na 2s, Na 1s, Na 2p, Ca 2s, O 1s, Na KLL and O KLL as shown in Fig. 16di. The survey spectrum of GO shows the presence of C 1s, O 1s and O KLL as shown in Fig. 16ei. The Si 2p spectra assigned two peaks for BG/GO at 104.67 and 102.63 eV and 102.5 eV for BG (Na-free)/GO as SiO_2_ and Si 2p_3/2_ state (Fig. 16aii,bii). The Ca 2p spectra for BG/GO display three peaks at 350.97, 348.2, and 347.15 eV for Ca 2p_1/2_, CaCO_3_, and Ca(PO_4_)_2_ (Fig. 16aiii). Further, two peaks at 350.57 and 347 eV assigned to Ca 2p_1/2_ and Ca 2p_3/2_ for BG (Na-free)/GO (Fig. 16biii). The P 2p spectra revealed two peaks at 134.6 and 133.1 eV for the P 2p_3/2_ state and PO_3_ formation for BG/GO, and a single peak at 133.5 eV for the P 2p_3/2_ state for BG (Na-free)/GO^52^ (Fig. 16aiv,biv). The C 1s spectra of BG/GO reveal three peaks at 290, 286.4, and 284.7 eV, and two peaks at 289.3 and 284.7 eV for BG (Na-free)/GO, with C=O, C–O–C, and C–C bonds attributed to the peaks (Fig. 16avi,bv). The high-resolution spectrum of O 1s shows two peaks at 532.7 and 531.3 eV for BG/GO and 532.7 and 531.2 eV for BG (Na-free)/GO ascribed to oxygen-silica and oxygen-carbon bonding, respectively (Fig. 16avii,bvi). The Na 1s showed two peaks for Na–O, and (Na–O–Si NBO) bonds at 1073.4 and 1072 eV^53,54^ (Fig. 16av). In GO, the C1s spectrum contains three peaks at 284.7, 286.9, and 285.6 eV, corresponding to the sp^2^ carbon, the epoxide, carboxyl, and carbonyl functional group two Si 2p spectra for BG show lower and higher energy shifts (101.5 and 103.1 eV). The carbon peak at 284.8, 286.8, and 288.9 eV was assigned for carboxyl groups, and the presence of CaCO_3_ increased with GO composition (Fig. 16eii). The oxygen spectrum of GO at 532.93 eV is assigned as C=O, C–O–C (Fig. 16eiii). The BG (Na-free) shows SiO_2_ peak at 102.96 eV and Si 2p_3/2_ peak at 101.3 eV and BG shows SiO_2_ peak at 103.18 eV and Si_3_N_4_ peak at 101.53 eV (Fig. 16cii,dii). The Ca 2p state as two peaks of Ca 2p_3/2_ and Ca 2p_1/2_ for BG (Na-free) at 347.6 and 251.2 eV and for BG at 347.04 and 350.7 eV (Fig. 16ciii,diii). The BG (Na-free) and BG shows phosphours peaks of P 2p_3/2_, PO_3_ and P–C at 133.7, 133.15 and 131.79 eV respectively (Fig. 16civ,div). For BG(Na-free) the Na 1s peak occurs at 1072. eV and 1071.14 eV for Na–O (Fig. 16dv). The high-resolution spectrum of O 1s shows one peak for BG (Na-free) at 532.7 eV as O–Si and three peaks for BG at 531.17, 532.82 and 535.6 eV as O–Si, Bridging oxygen and Si–O–Si respectively (Fig. 16cv,dvi). The results show that the relative positions of XPS peaks are associated with P–O bonding, CaCO_3_ formation, and Si 2p_3/2_ at a stable state with a GO matrix.

#### Structural defects and functional group examination

The Raman spectra of BG, BG (Na-free), GO, and BG/GO, BG (Na-free)/GO nanocomposites are displayed in Supplementary Fig. S6. The D and G bands of pure GO correspond to structural disorder, and the stretching of the carbon sp^2^ bond with the I_D_/I_G_ ratio is ~ 0.9. The incorporation of GO with 45S5 BG results in a decrease in the intensity of both D and G bands and a hypsochromic shift ~ 1343 and 1566 cm^−1^, indicating that GO caused a surface attachment on the 45S5 BG matrix. The band peaks at ~ 947, 986, and 594 cm^−1^ suggest P–O symmetrical stretching, P–O–P stretching, and P–O bending vibrations, respectively. The non-bridging oxygen sites in the Si–O–Si bond with monomers of SiO_4_^4−^ correspond at 868 cm^−1^.

Fourier transform infrared (FTIR) studies were used to assess the apatite layer deposition on the BG, BG (Na-free), GO and BG/GO, BG (Na-free)/GO nanocomposite surfaces (Supplementary Fig. S7a). The bands appeared at ~ 1439, 1027, 759, 570, and 457 cm^−1^ attributed to the (C–O) residual, (Si–O–C) (stretch), SiO_4_, PO_4_, (Si–O–Si) stretch, and P–O (bend), respectively. The signal at ~ 1027 cm^-1^ shifts to (Si–O–C) asymmetric stretching vibration, indicating that the GO carbonyl group has been transformed to (Si–O–C) with the assistance of the 45S5 BG SiO_2_ group^55^. In addition, the mineralized samples (Supplementary Fig. S7b–d) have in-plane stretching vibrations of C–H, C–N, and C–O at 2984, 2854, and 1458 cm^−1^, respectively, indicating the creation of the CHAp phase and the detailed vibrational planes were shown in Supplementary Table S4. The change in (Si–O–Si) stretching and bending vibrations around 1028 to 1018 cm^−1^ and 472 to 455 cm^−1^ indicated apatite layer formation. By establishing calcium carbonate and phosphate areas, the Si–O–Si stretching band at 1028 cm^−1^ becomes less intense as the mineralization days increases. Hence, the FTIR findings for apatite production validated the studied composite material’s intermolecular interaction.

##### Thermal studies

The BG and BG (Na-free) samples were subjected to TG/DTA analyses before annealing and presented in Supplementary Fig. S8a,b at different intervals. The endothermic peak in BG is associated with a peak at 135 °C and the elimination of H_2_O molecules, while the TG curve reveals an 11.26% weight loss at 200 °C. At 567 and 306 °C, the second weight loss endothermic peak of 18.98% occurs, further increasing the destruction of organic molecules. Similarly, from 134 to 570 °C, the BG (Na-free) displays a total weight loss of 37.7%, with DTA maxima at 194, 300, and 570 °C determining the endothermic process.

#### Mechanical stability analysis

The microhardness test was obtained at room temperature with an applied load of 0.3 Kgf and a dwell time of 10 s which shown in Supplementary Fig. S9. All the samples are indicated with error bars and denote a decrease in hardness when load increases. Compared to pure 45S5 BG and GO, the nanocomposites show a linear increase in hardness due to the microstructure of the GO.

## Conclusion

The in vitro biological efficacy of BG, BG (Na-free), GO and BG/GO, and BG (Na-free)/GO nanocomposites were extensively investigated. The hemoclot experiment indicates that BG (Na-free) can clot blood in 3 min, an essential step in wound healing. The interlink between BG (Na-free) and GO and their nanocomposites had an increased fracture toughness of nearly 85%. An in vitro wound healing test of the BG, BG (Na-free), GO, and BG/GO, BG (Na-free)/GO nanocomposites demonstrate 30–40% wound healing up to 24 h. In which, at greater concentrations, the BG (Na-free) and BG exhibit improved wound closure, but the BG (Na-free)/GO, BG/GO, and GO do not show meaningful effects because the GO nanosheets may hinder cell growth. The generation of oxygen species and hydroxyl radicals in 45S5 BG and GO materials was measured using the DPPH test, with an effective scavenging performance of 80% for BG (Na-free), 60% for BG, 65% for BG (Na-free)/GO, 80% for BG/GO, and 70% for GO at higher concentrations (70 mg/mL). BG and BG (Na-free) composites performed better in the early stages of wound healing, whereas the GO composites had more significant stability and anti-inflammatory properties. Collectively, our findings suggest that 45S5 BG and GO nanocomposites are potential materials for chronic or diabetic wound healing in the early stages. The proposed nanocomposites will be used to fabricate wound healing pads, bandages, or sheets as a product with in vivo models shortly.

### Materials and methods

TEOS (Si(OC_2_H_5_)_4_) TCL, orthophosphoric acid (H_3_PO_4_) Merck, calcium nitrate (CaNO_3_), sodium nitrate (NaNO_3_), sulphuric acid (H_2_SO_4_), hydrochloric acid (HCl), and sodium nitrate (NaNO_3_) SRL, hydrogen peroxide (H_2_O_2_) Merck, potassium permanganate (KMnO_4_) SRL, nitric acid (HNO_3_) SRL, graphite (Sigma), Cell-Line L929 mouse fibroblast cells, NCCS Pune. XRD (X-Pert Pro PANalytical, Netherlands), HRTEM (Jeol/JEM 2100, Japan), SEM (Hitachi S-3400N, Japan), XPS (Physical Electronics), FTIR (SHIMADZU (Miracle 10, US), RAMAN (WiTec alpha 300, Germany), TGA (NETZSCH STA 2500, Germany), UV–Visible Spectroscopy (LAB MAN LMSP-UV1200, India), Hardness (Wilson-Wolpert, Canada), ICP-OES (Perkin Elmer Optima 5300DV, USA), ELISA Plate Analyser (ROBONIK).

### Preparation of 45S5 bioglass (45S5 BG)

The 45S5 BG was synthesized by the sol–gel method with a composition of 46.1% SiO_2_, 26.9% CaO, 24.4% Na_2_O, and 2.6 P_2_O_5_ precursors with the stoichiometry of 11.48 mL tetraethyl ortho silicate (TEOS), 1.74 mL H_3_PO_4_, 6.24 g of CaNO_3,_ and 13.84 g of NaNO_3_ as shown in Supplementary Fig. S10^56,57^. All the precursors were prepared in double-distilled (DD) water. TEOS + HNO_3_ was first dissolved in ethanol and DD water (1:2) suspension and held under constant stirring. The suspension is allowed to form gel until it reaches a clear solution. After the gel was formed, H_3_PO_4_ was added dropwise to the agitated solution until it dissolved entirely, and subsequently, the gelation process was allowed further for 12 h. The CaNO_3_ was then added dropwise and kept at 80 °C for 36 h. The samples were also dried for 24 h in a hot air oven at 100 °C. The as-prepared samples were calcined for 3 h at 800 °C (heating rate 5 °C/min) to achieve phase formation without residues and were designated as BG. Similarly, the 45S5 bioglass was made without sodium and is termed BG (Na-free) with a similar synthesis process.

### Synthesis of graphene oxide (GO)

To avoid environmental concerns, the revised hammers process uses H_3_PO_4_ instead of NaNO_3_, which is non-toxic and produces high-quality GO sheets^58,59^. The improved hammers approach was used to synthesize the GO samples as shown in Supplementary Fig. S11. 2.0 g graphite powder was typically added to 100 mL of saturated H_2_SO_4_ under constant stirring followed by dropwise H_3_PO_4_ addition. The H_2_SO_4_:H_3_PO_4_ ratio was calculated to be 100:1. The KMnO_4_ (6.0 g) was progressively added for exploitation while stirring continuously with a cold bath. The diluted liquid became brown after complete exfoliation, indicating hydrolysis and absolute exfoliation of intercalated graphite oxide. The brown suspension was then turned black with the addition of H_2_O_2_ to remove the remaining oxidants. Finally, the resulting solution was washed numerous times with HCl and DD water to neutralize the pH and eliminate excess residuals. The washed and filtered samples were dried for 24 h at 100 °C.

#### Preparation of BG-GO nanocomposite

A simple reduction approach employing hydrazine hydrate and probe- and ultra-sonication methods were used to fabricate the BG and GO as a nanocomposite as shown in Supplementary Fig. S12. The BG and BG (Na-free) samples were weighed (900 mg), dispersed in 10 mL DD water, and stirred for 1 h. The BG and GO suspensions were mixed and maintained under constant stirring. The GO granules (100 mg) were added slowly and probe sonicated for 15 min. 15 mL hydrazine hydrate was introduced to reduce the oxygen molecule in GO and further ultra-sonicated for 2–3 h. Finally, sonicated samples were washed, filtered, and then dried in a vacuum oven at 100 °C for 12 h. The prepared samples were named BG/GO nanocomposite (BG with Na) and BG (Na-free)/GO nanocomposite (BG without Na).

### In vitro biomineralization assay

This investigation employed Hank’s balanced salt solution as the simulated body fluid (SBF) solution. Na^+^ 213.0 mM, K^+^ 7.5 mM, Mg^2+^ 2.25 mM, Ca^2+^ 3.75 mM, Cl^-^ 221.7 mM, HCO_3_^−^ 6.3 mM, HPO_4_^2−^ 1.5 mM, SO_4_^2-^ 0.75 mM are the components of SBF. The BG, BG (Na free), GO and BG/GO, BG (Na free)/GO nanocomposite pellets were incubated at 37 °C for up to 28 days in 10 mL SBF (pH 7.4) in 15 mL falcon tubes^60^. The submerged samples were washed extensively with DD water and dried at 100 °C under a vacuum. XRD, FTIR, Raman, and SEM investigations were carried out to examine the development of apatite layer formation on composite surfaces.

### In vitro biocompatibility assay

Healthy volunteers provided the appropriate amount of blood, mixed with 1.6 g/L of ethylenediaminetetraacetic acid (EDTA) to avoid coagulation. The blood was centrifuged at 4 °C for 10 min at 2000 rpm. Phosphate buffer saline (PBS) was used to wash the collected erythrocytes (RBCs). The RBCs were then resuspended in a suitable amount of PBS (pH 7.4). The positive control comprises 950 μL of DD water and 50 μL of blood, whereas the negative control has 950 μL of PBS and 50 μL of blood. A calculated number of samples were placed in a vial containing 1 mL of re-suspended RBCs and incubated for 1 h at 37 °C with gentle shaking. The vials were then centrifuged for 10 min at 2000 rpm, and the supernatant was collected. Finally, using a UV–Visible Spectrophotometer, the absorbance was measured at 540 nm. The following equation (1) was used to compute the percentage of hemolysis;

1 $$\left[ {{\text{Hemolytic percentage }} = \, \frac{{{\text{sample absorbance }} - {\text{ negative control}}}}{{{\text{positive control }} - {\text{ negative control}}}} \times 100} \right].$$

### In vitro cytotoxicity and cell proliferation MTT assay-direct method

A direct Methyl thiazolyl diphenyl tetrazolium bromide (MTT) test was used to investigate the cytotoxicity of BG and GO. The fibroblast L929 cells were grown in Dulbecco’s Modified Eagle’s medium (DMEM), which included 10% fetal bovine serum (FBS) and 1% penicillin-streptomycin^61^. The cells (1 × 10^5^ per well) were planted in 24-well plates and incubated at 37 °C with 5% CO_2_. The cells were treated with various concentrations of all samples (0, 0.5, 2.5, 5.0, 7.5, 10.0 μM) for 24 and 48 h to assess the cytotoxicity based on the concentration of the samples. The mitochondrial enzyme decrease was achieved by adding 5 mg/mL of MTT to the samples and incubating them for 4 h. Following the incubation period, 1 mL of dimethyl sulfoxide (DMSO) was added to each well to dissolve the formazan crystals^62^. The absorbance at 570 nm was measured using an ELISA reader with DMSO as a control. Measurements found the concentration necessary for a 50% inhibition (IC_50_). An inverted phase-contrast microscope was also used to view the morphology of the cells. The percentage of viable cells was computed using the following Eq. (2),

2 $${\text{Cell viability }}\left( \% \right) \, = \, \left( {{\text{Corrected abs. of sample}}/{\text{corrected abs. of control}}} \right) \, \times { 1}00.$$

### Apoptosis assay

The development of apoptosis is a key element of wound healing since it influences cell function and cytocompatibility. Exponentially developed L929 cells were planted in 48-well plates with a cell density of 50,000 cells per well for apoptosis tests. The cell lines were grown at 37 °C with 5% CO_2_ in Minimum Essential Medium Eagle (MEM) containing 10% fetal bovine serum (FBS) and a Penicillin (100 U/mL)–Streptomycin (100 g/mL) antibiotic solution^63^. After alcoholic cell fixation, the cells were stained with 50 μL of double dye (Acridine orange/Ethidium bromide) and 10 μL of a medium, which was allowed to bind for 10 min before the media was withdrawn. The cells were cultured with samples (0.1 mg/mL) at varying concentrations (0, 5, 25, 50, 75, 100 μL) for 24 and 48 h at 37 °C after the medium was withdrawn and rinsed with PBS buffer for the experimental groups. A fluorescence microscope was used to capture the fluorescence pictures in the UV-region, blue channel.

### Wound healing (scratch assay)

For cell migration studies, the scratch assay was developed. The fibroblast L929 cells were cultured in DMEM with 10% FBS supplemented. After the cells had grown to roughly 80% confluence in a 24-well tissue culture plate for 24 h, a slight scratch was formed on the monolayer of the cells using a fresh 1 mL pipette tip across the well. Scratched wells were gently washed to remove the detached cells. The BG, BG (Na free), GO and BG/GO, BG (Na free)/GO nanocomposites were added to the wells (0.1 mg/mL), and the cells were left to heal for the period allotted. In addition, using an inverted phase-contrast microscope, time-dependent pictures were acquired. Finally, using Eq. (3), the wound contraction % was computed.

3 $$\left[\text{\% Wound Contraction}= \frac{{\text{Wound area at initial hours}}-\text{ Wound area at final hour}}{\text{Wound area at initial hour}}\times 100\right].$$

### Antioxidant activity

Generally, the scavenging activity of antioxidants is measured using 1,1-DPPH, a synthetic nitrogen-centered free radical, that accepts electrons or hydrogen radicals from antioxidants to form a stable molecule. The free radical scavenging activity of nanocomposites was assessed by using the DPPH assay. The BG (Na free), BG, GO, BG (Na free)/GO, and BG/GO were immersed in 2 mL of methanol solution overnight. Then, 2.5 mL of DPPH radical solution (concentration of 0.04 mg/mL) was added to 0.5 mL of methanol solution and the sample. After 30 min of incubation in the dark at 37 °C, the absorbance was measured using UV–Visible spectroscopy at 517 nm. The blank and control solutions were methanol and DPPH, respectively. The following equation was used to compute DPPH radical scavenging activity^64^.

$${\text{DPPH radical scavenging activity }}\left( \% \right) \, = \frac{{{\text{Absorbance of control}} - {\text{Absorbance of sample}}}}{{\text{Absorbance of control}}} \times 100$$

### Antibacterial assay

The antibacterial activity was characterized via the colony counting method. *S. aureus* was used to represent gram-positive and *P. aeruginosa* as gram-negative bacteria. Bacteria were inoculated individually with 20 mL of Luria–Bertani (LB) broth media. After a revival of the culture, a loop full of culture was introduced into the sterile fresh broth and incubated at 37 °C in an orbital shaker. After reaching the mid-log phase, the cells were centrifuged (4 °C, 4000 rpm for 10 to 15 min), then obtained pellets were washed several times using PBS. Further, the cells were used to analyze the antibacterial activity in the presence of materials. The samples were well sonicated using a bath sonicator, and the ratio of the samples (1 mg) with PBS, sterile broth, and cell suspension used to be at 8:1:1. Then the materials were incubated with the presence of bacteria for 6 h. Thereafter serially diluted to 10 dilutions, after that, 10^−5^, 10^−6^, and 10^−7^ dilutions were smeared into the agar poured, prepared plates Mueller–Hinton (MH) agar. Furthermore, the plates were incubated for overnight to count the minimum inhibitory concentration (MIC), in which bacteria without material is used as a negative control. Similarly, MH agar poured plates were used to enumerate the zone of inhibition, and wells were created in the microbial species spreader plates. Antibiotic (tetracycline) is used as a positive control the remaining four wells were used to introduce the samples of 20 mg/mL. After overnight incubation, the zone was captured through a camera and MIC photographic images corresponding to 10^−7^ dilutions were exhibited^65^.

### Statistical analysis

To compare the means of more than two groups, statistical analysis was performed using the standard one-way analysis of variance with significant levels. Statistical analysis Data were analyzed by ANOVA and Bonferroni’s test using Origin (Origin Lab, Northampton, MA, USA).

## Supplementary Information

Supplementary Information.

## Data Availability

All data generated or analyzed during this study are included in this published article (and its Supplementary Information files).

## References

[1] Xie H, Sha S, Lu L, Wu G, Jiang H, Boccaccini AR, Zheng K, Xu R (2022). Cerium-containing bioactive glasses promote in vitro Lymphangiogenesis. Pharmaceutics.

[2] Armstrong DG, Galiano RD, Glat PM, DiDomenico LA, Carter MJ, Zelen CM (2022). A multi-centre, single-blinded randomised controlled clinical trial evaluating the effect of resorbable glass fibre matrix in the treatment of diabetic foot ulcers. Int. Wound J..

[3] Kargozar S, Hamzehlou S, Baino F (2019). Can bioactive glasses be useful to accelerate the healing of epithelial tissues?. Mater. Sci. Eng. C.

[4] Saravanan S, Chawla A, Vairamani M, Sastry TP, Subramanian KS, Selvamurugan N (2017). Scaffolds containing chitosan, gelatin and graphene oxide for bone tissue regeneration in vitro and in vivo. Int. J. Biol. Macromol..

[5] Siti Fatimah S, Siti Noor Fazliah MN, Mislia O, Nur Syazana A, Muhammad Azrul Z (2020). Development of bioactive glass-poly-Ɛ-caprolactone polymer composite film for soft tissue regeneration. AIP Conf. Proc..

[6] Rizwan M, Hamdi M, Basirun WJ (2017). Bioglass^®^ 45S5-based composites for bone tissue engineering and functional applications. J. Biomed. Mater. Res. A.

[7] Heng MCY (2011). Wound healing in adult skin: Aiming for perfect regeneration. Int. J. Dermatol..

[8] Oh H, Kim CH, Lee YJ (2022). Bullous pemphigoid diagnosis: The role of routine formalin-fixed paraffin-embedded skin tissue immunochemistry. Sci. Rep..

[9] Byrd A, Belkaid Y, Segre J (2018). The human skin microbiome. Nat. Rev. Microbiol..

[10] Jones J, Brauer D, Hupa L, Greenspan D (2016). Bioglass and bioactive glasses and their impact on healthcare. Int. J. Appl. Glass Sci..

[11] Hench LL (2006). The story of bioglass®. J. Mater. Sci. Mater. Med..

[12] Kovacs Z, Fabian M, Szasz M, Szekacs I, Kovacs Kis V (2022). Tracking the initial stage of bioactive layer formation on Si-Ca-Na-P oxide glasses by nanoindentation. J. Non-Cryst. Solids.

[13] Zhou H, Liang B, Jiang H, Deng Z, Yu K (2021). Magnesium-based biomaterials as emerging agents for bone repair and regeneration: From mechanism to application. J. Magnes. Alloys.

[14] Kargozar S, Singh RK, Kim HW, Baino F (2020). “Hard” ceramics for “soft” tissue engineering: Paradox or opportunity?. Acta Biomater..

[15] Fiume E, Barberi J, Verne E, Baino F (2018). Bioactive glasses: From parent 45S5 composition to scaffold-assisted tissue-healing therapies. J. Funct. Biomater..

[16] Shuai W, Xiaoyang C, Xiaomu X, Jiacheng W, Zhiqiang G, Zhenzhao G, Pinh H, Changren Z, Hong L (2022). In vivo and in vitro evaluation of chitosan-modified bioactive glass paste for wound healing. J. Mater. Chem. B.

[17] Erol O, Uyan I, Hatip M, Yilmaz C, Tekinay AB, Guler MO (2018). Recent advances in bioactive 1D and 2D carbon nanomaterials for biomedical applications. Nanomed. Nanotechnol. Biol. Med..

[18] Hu W, Peng C, Luo W, Lv M, Li X, Li D, Huang Q, Fan C (2010). Graphene-based antibacterial paper. ACS Nano.

[19] Muhammad SA, Muhammad SA, Ahmad MA (2017). Application of graphene, graphene oxide and their derivatives as Wound healing: A brief review. Adv. Environ. Biol..

[20] Weaver CL, Cui XT (2015). Directed neural stem cell differentiation with a functionalized graphene oxide nanocomposite. Adv. Healthc. Mater..

[21] Shin SR, Zihlmann C, Akbari M, Assawes P, Cheung L, Zhang K, Manoharan V, Zhang YS, Yuksekkaya M, Wan KT, Nikkhah M, Dokmeci MR, Tang XS, Khademhosseini A (2016). Reduced graphene oxide-GelMA hybrid hydrogels as scaffolds for cardiac tissue engineering. Small.

[22] Whitelock, J. *Overview of Necrosis in Human Body* (2021)

[23] Chen X, Chen X, Brauer DS, Wilson RM, Law RV, Hill RV, Karpukhina N (2017). Sodium is not essential for high bioactivity of glasses. Int. J. Appl. Glass. Sci..

[24] Dey P (2020). Bone Mineralization.

[25] Hwang ZA, Suh KJ, Chen D, Chan WP, Wu JS (2018). Imaging features of soft-tissue calcifications and related diseases: A systematic approach. Korean J. Radiol..

[26] Murshed M, Kempf H, Komarova S (2021). Ectopic Mineralization of Tissues: Mechanisms, Risk Factors, Diseases and Prevention.

[27] Chen QZ, Thouas GA (2011). Fabrication and characterization of sol-gel derived 45S5 bioglass-ceramic scaffolds. Acta Biomater..

[28] Xie J, Blough ER, Wang CH (2012). Submicron bioactive glass tubes for bone tissue engineering. Acta Biomater..

[29] Cini M, Legnani C, Cosmi B, Testa S, Dellanoce C, Paoletti O, Marcucci R, Poli D, Paniccia R, Pengo V, Tripodi A, Palareti G (2018). Comparison of five specific assays for determination of dabigatran plasma concentrations in patients enrolled in the START-Laboratory Register. Int. J. Lab. Hematol..

[30] Kalaria T, Gill H, Harris S, Ford C, Gama R (2021). The effect of haemolysis on the direct and indirect ion-selective electrode measurement of sodium. Ann. Clin. Biochem..

[31] Belling JN, Jackman JA, Yorulmaz Avsar S, Park JH, Wang Y, Potroz MG, Ferhan AR, Weiss PS, Cho NJ (2016). Stealth immune properties of graphene oxide enabled by surface-bound complement factor H. ACS Nano.

[32] de Brito Sousa JD, Sachett JAG, de Oliveira SS, Mendonça-da-Silva I, Marques HO, de Lacerda MVG, Fan HW, Monteiro WM (2018). Accuracy of the lee-white clotting time performed in the hospital routine to detect coagulopathy in *Bothrops atrox* envenomation. Am. J. Trop. Med. Hyg..

[33] Sneha S, Johannes D, Peter V, Emma H, Helen P, Vytautus I, Diana I, Johannes O, Arijit B (2019). Structure functional insights into calcium binding during the activation of coagulation factor XIII A. Sci. Rep..

[34] Huntington JA (2008). How Na^+^ activates thrombin—A review of the functional and structural data. Biol. Chem..

[35] Sarode DN, Roy S (2019). In vitro models for thrombogenicity testing of blood-recirculating medical devices. Expert Rev. Med. Devices.

[36] Wallace KE, Hill RG, Pembroke JT, Brown CJ, Hatton PV (1999). Influence of sodium oxide content on bioactive glass properties. J. Mater. Sci. Mater. Med..

[37] Echezarreta-Lopez M, Landin M (2013). Using machine learning for improving knowledge on antibacterial effect of bioactive glass. Int. J. Pharm..

[38] Hu S, Chang J, Liu M, Ning C (2009). Study on antibacterial effect of 45S5 bioglass. J. Mater. Sci. Mater. Med..

[39] Saxena S, Tyson TA, Shukla S, Negusse E, Chen H, Bai J (2011). Investigation of structural and electronic properties of graphene oxide. Appl. Phys. Lett..

[40] Liu X, Sen S, Liu J, Kulaots I, Geohegan D, Kane A, Puretzky AA, Rouleau CM, More KL, Palmore GTR, Hurt RH (2011). Antioxidant deactivation on graphenic nanocarbon surfaces. Small.

[41] Sudhin T, Vignesh M, Ramesh P (2015). Mechanical characterization of high-performance graphene oxide incorporated aligned fibroporous poly(carbonate urethane) membrane for potential biomedical applications. J. Appl. Polym. Sci..

[42] Xynos DI, Hukkanen MVJ, Batten JJ, Buttery LD, Hench LL, Polak JM (2000). Bioglass 45S5 stimulates osteoblast turnover and enhances bone formation in vitro: Implications and applications for bone tissue engineering. Calcif. Tissue. Int..

[43] Deng L, Du C, Song P, Chen T, Rui S, Armstrong DG, Deng W (2021). The role of oxidative stress and antioxidants in diabetic wound healing. Oxid. Med. Cell. Longev..

[44] Sanchez MC, Lancel S, Boulanger E, Neviere R (2018). Targeting oxidative stress and mitochondrial dysfunction in the treatment of impaired wound healing: A systematic review. Antioxidants (Basel).

[45] Chaniad P, Tewtrakul S, Sudsai T, Langyanai S, Kaewdana K (2020). Anti-inflammatory, wound healing and antioxidant potential of compounds from *Dioscorea bulbifera* L. bulbils. PLoS ONE.

[46] Fu R, Zhang YT, Guo YR, Huang QL, Peng T, Xu Y, Tang L, Chen F (2013). Antioxidant and anti-inflammatory activities of the phenolic extracts of *Sapium sebiferum* (L.) Roxb. leaves. J. Ethnopharmacol..

[47] Rajeswari R, Prabu HG (2018). Synthesis characterization, antimicrobial, antioxidant, and cytotoxic activities of ZnO nanorods on reduced graphene oxide. J. Inorg. Organomet. Polym..

[48] Hamam KA, Gaabour LH (2017). Verification of the changes in the structural and physical properties of PU/PEO embedded with graphene oxide. Results Phys..

[49] Liang B, Liu K, Liu P, Qian L, Zhao G, Pan W, Chen C (2021). Organic salt-assisted liquid-phase shear exfoliation of expanded graphite into graphene nanosheets. J. Materiomics.

[50] Bargavi P, Ramya R, Chitra S, Vijayakumari S, Riju Chandran R, Durgalakshmi D, Rajashree P, Balakumar S (2020). Bioactive, degradable and multi-functional three-dimensional membranous scaffolds of bioglass and alginate composites for tissue regenerative applications. Biomater. Sci..

[51] Mackovic M, Hoppe A, Detsch R, Mohn D, Stark WJ, Spiecker E, Boccaccini AR (2012). Bioactive glass (type 45S5) nanoparticles: In vitro reactivity on nanoscale and biocompatibility. J. Nanopart. Res..

[52] Hart JN, May PW, Allan NL, Hallam KR, Claeyssens F, Fuge GM, Ruda M, Heard PJ (2013). Towards new binary compounds: Synthesis of amorphous phosphorus carbide by pulsed laser deposition. J. Solid State Chem..

[53] Kalapsazova ML, Zhecheva EN, Tyuliev GT, Nihtianova DD, Mihaylov L, Stoyanova RK (2017). Effects of the particle size distribution and the electrolyte salt on the intercalation properties of P3-Na_2/3_Ni_1/2_Mn_1/2_O_2_. J. Phys. Chem. C.

[54] Nesbitt HW, Bancroft GM, Henderson GS, Ho R, Dalby KN, Huang Y, Yan Z (2011). Bridging, non-bridging and free (O^2–^) oxygen in Na_2_O-SiO_2_ glasses: An X-ray photoelectron spectroscopic (XPS) and nuclear magnetic resonance (NMR) study. J. Non-Cryst. Solids.

[55] Wang W, Liu Y, Yang C, Qi X, Li S, Liu C, Li X (2019). Mesoporous bioactive glass combined with graphene oxide scaffolds for bone repair. Int. J. Biol. Sci..

[56] Ashok Raja C, Balakumar S, Bargavi P, Rajashree P, Anandkumar B, George RP, Kamachi Mudali U (2018). Decoration of 1-D nano bioactive glass on reduced graphene oxide sheets: Strategies and in vitro bioactivity studies. Mater. Sci. Eng. C.

[57] Kumar A, Sevi M, Aditya A, Boccaccini AR (2017). Mesoporous 45S5 bioactive glass: Synthesis, in vitro dissolution and biomineralization behaviour. J. Mater. Chem. B.

[58] Yu H, Zhang B, Bulin C, Li R, Xing R (2016). High-efficient synthesis of graphene oxide based on improved hummers method. Sci. Rep..

[59] Zineb B, Pengwan C, Levent T (2021). Enhanced synthesis method of graphene oxide. Nanoscale Adv..

[60] Liu H, Cheng J, Chen F, Bai D, Shao C, Wang J, Xi P, Zeng Z (2014). Gelatin functionalized graphene oxide for mineralization of hydroxyapatite: Biomimetic and in vitro evaluation. Nanoscale.

[61] Rugia Abu, A. M. M. A. & Alhassan. *Investigating the Role of Mineralization of Bioactive Glass in Wound Healing Using a Cellular 3D Collagen Matrix*, *Thesis* (McGill University, 2020).

[62] Chitra S, Bargavi P, Riju Chandran R, Balakumar S (2020). Exploration of thermal treatment dependent in vitro mineralization on 45S5 bioactive nanostructured materials. AIP Conf. Proc..

[63] Zhang G, Gurtu V, Kain SR, Yan G (1997). Early detection of apoptosis using a fluorescent conjugate of annexin V. Biotechniques.

[64] Irem U, Fuggerer T, Benedikt S, Andrea B, Boccaccini AR (2021). Antibacterial and antioxidant activity of cinnamon essential oil-laden 45S5 bioactive glass/soy protein composite scaffolds for the treatment of bone infections and oxidative stress. Mater. Sci. Eng. C.

[65] Chitra S, Bargavi P, Balasubramaniam M, Riju Chandran R, Balakumar S (2020). Impact of copper on in vitro biomineralization, drug release efficacy and antimicrobial properties of bioactive glasses. Mater. Sci. Eng. C.

