# Supplementary material for: An enduring in vitro wound healing phase recipient by bioactive glass-graphene oxide nanocomposites
Source: Sci Rep. 2022 Sep 28;12:16162. doi: 10.1038/s41598-022-20575-z (PMC9519557; doi:10.1038/s41598-022-20575-z)
Supplement: Supplementary file 1 — Supplementary Information. [file 41598_2022_20575_MOESM1_ESM.doc]

**Supplementary Information**

**An Enduring *In vitro* Wound Healing Phase Recipient by Bioactive Glass-Graphene Oxide Nanocomposites**

Manjubaashini Nandhakumar^1^, Daniel Thangadurai Thangaian^2^, Senthilarasu Sundaram^3*^, Anurag Roy^4^, Balakumar Subramanian^1*^

^1^National Centre for Nanoscience and Nanotechnology, University of Madras, Chennai-600 025, India.

^2^Department of Chemistry, KPR Institute of Engineering and Technology, Coimbatore-641 407, India.

^3^Electrical and Electronics Engineering, School of Engineering and the Built Environment, Edinburgh Napier University, Edinburgh, EH10 5DT, U.K.

^4^Environment and Sustainable Institute, University of Exeter, Penryn Campus, Cornwall TR10 9FE, UK.

**Corresponding Authors Email. Id:** [balasuga@yahoo.com](mailto:balasuga@yahoo.com) (Dr. S.B), [S.Sundaram@napier.ac.uk](mailto:S.Sundaram@napier.ac.uk) (Dr. S.S).

**1. Result and Discussion**

**1.1 Elemental Analysis**


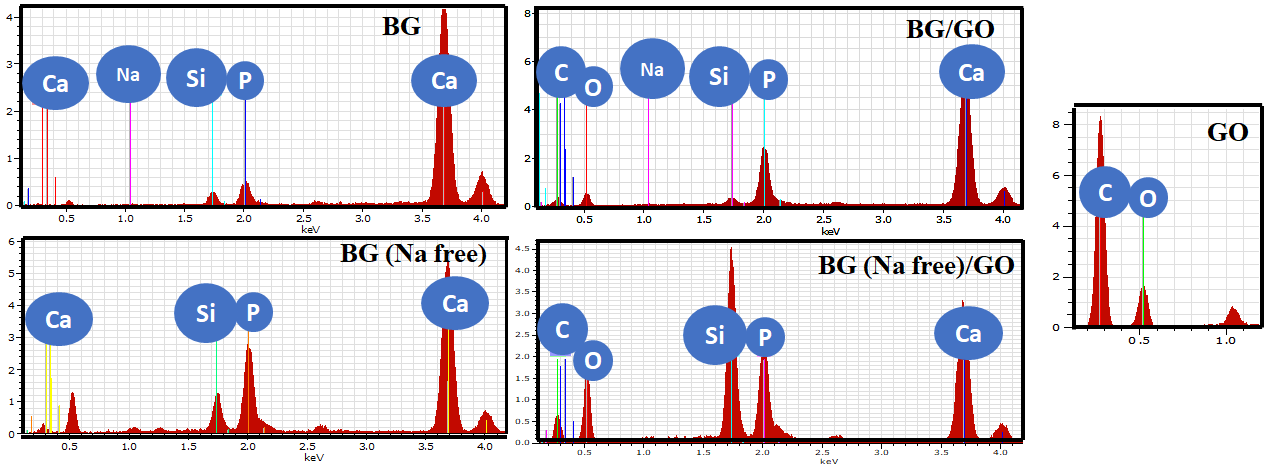


**Figure. S1.** EDAX spectrum of BG, BG (Na-free), GO and BG/GO, BG (Na-free)/GO nanocomposites after mineralization (28^th^ day).

**1.2 Hemoclot Assay**


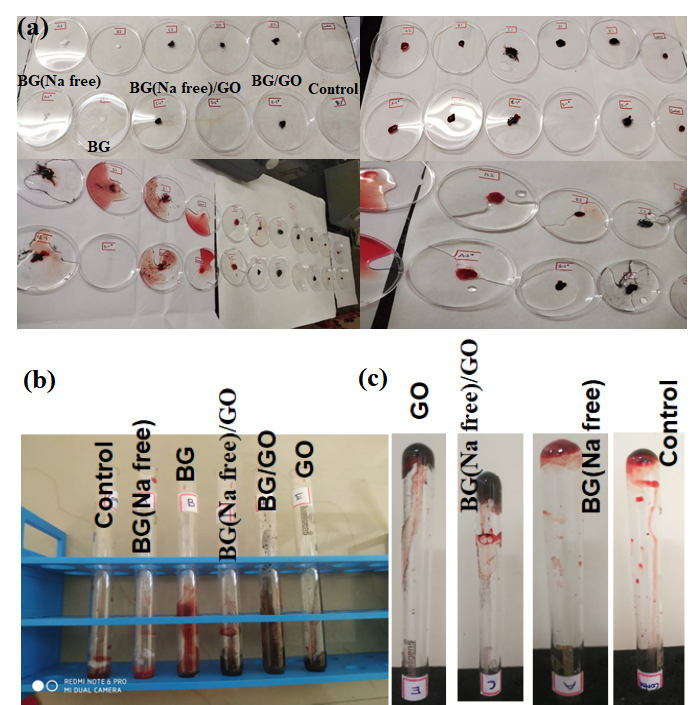


**Figure. S2.** (a) The whole blood interaction with samples, clot formation was disturbed by adding distilled water, (b) Le & white Method samples along with blood kept in a water bath at 37 °C for clot formation, (d) clot formation was confirmed by tilting the test tubes.

**1.3 Contact angle measurement**


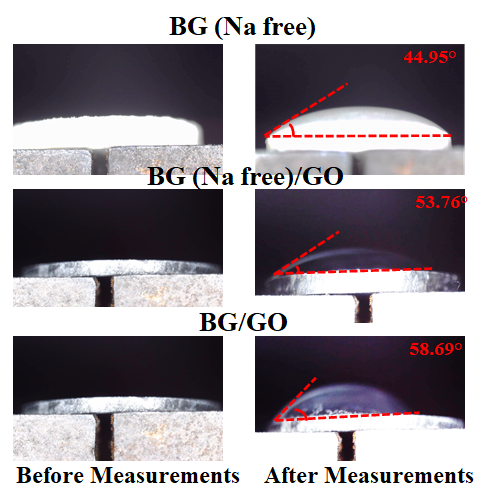


**Figure. S3.** Contact angle measurement of BG (Na-free), BG (Na-free)/(GO), and BG/GO.

**Table S1.** Peak parameters derived from Raman spectra.

| **Samples** | Before | **D band**  **(cm^-1^)** | **G band**  **(cm^-1^)** | **I_D_/I_G_** | **D´ band**  **(cm^-1^)** | **G´ band**  **(cm^-1^)** | **I´_D_/I´_G_** |
| --- | --- | --- | --- | --- | --- | --- | --- |
| GO |  | 1346 | 1591 | 0.90 | - | - | - |
|  | 1^st^ Day | 1328 | 1577 | 0.97 | 2687 | 2898 | 1.01 |
|  | 14^th^ Day | 1357 | 1599 | 0.98 | 2713 | 2936 | 0.98 |
|  | 28^th^ Day | 1357 | 1581 | 1.00 | 2700 | 2919 | 1.00 |
| BG/GO | Before | 1344 | 1569 | 0.45 |  |  |  |
|  | 1^st^ Day | - | - | - | - | - | - |
|  | 14^th^ Day | 1349 | 1578 | 1.03 | 2454 | 2714 | 0.89 |
|  | 28^th^ Day | 1352 | 1576 | 1.02 | 2710 | 2946 | 1.05 |
| BG (Na-free)/GO | Before | 1346 | 1594 | 1.14 | - | - | - |
| **Samples** | Before | **D band**  **(cm^-1^)** | **G band**  **(cm^-1^)** | **I_D_/I_G_** | **D´ band**  **(cm^-1^)** | **G´ band**  **(cm^-1^)** | **I´_D_/I´_G_** |
| GO |  | 1346 | 1591 | 0.90 | - | - | - |
|  | 1^st^ Day | 1328 | 1577 | 0.97 | 2687 | 2898 | 1.01 |
|  | 14^th^ Day | 1357 | 1599 | 0.98 | 2713 | 2936 | 0.98 |
|  | 28^th^ Day | 1357 | 1581 | 1.00 | 2700 | 2919 | 1.00 |
| BG/GO | Before | 1344 | 1569 | 0.45 |  |  |  |
|  | 1^st^ Day | - | - | - | - | - | - |
|  | 14^th^ Day | 1349 | 1578 | 1.03 | 2454 | 2714 | 0.89 |
|  | 28^th^ Day | 1352 | 1576 | 1.02 | 2710 | 2946 | 1.05 |
| BG (Na-free)/GO | Before | 1346 | 1594 | 1.14 | - | - | - |


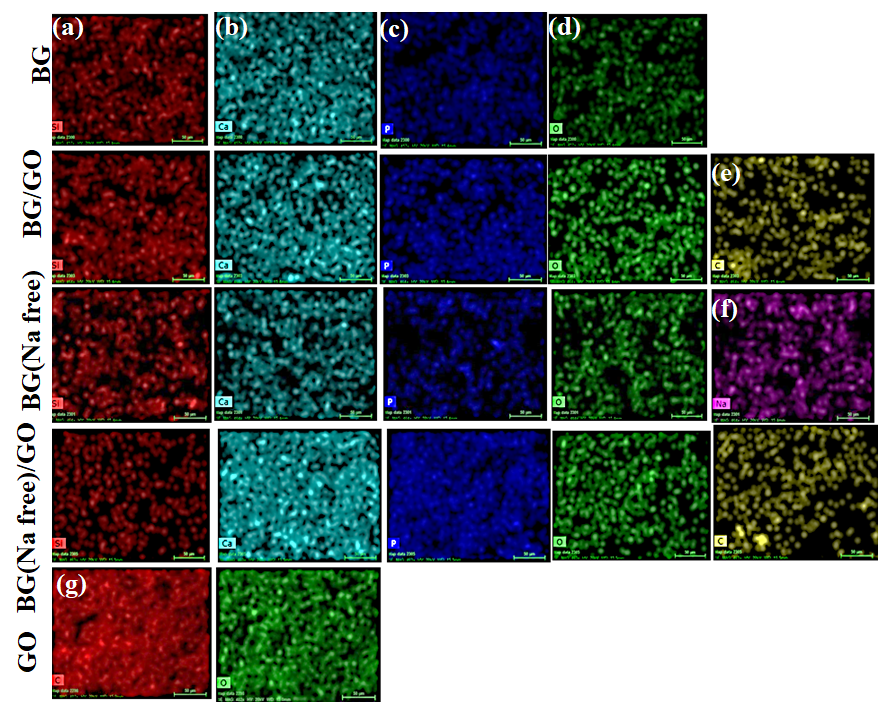


**Figure. S4.** Elemental mapping of BG, BG (Na-free), GO and BG/GO, BG (Na-free)/GO nanocomposites. (a) Si, (b) Ca, (c) P, (d) O, (f) Na, (e & g) C.


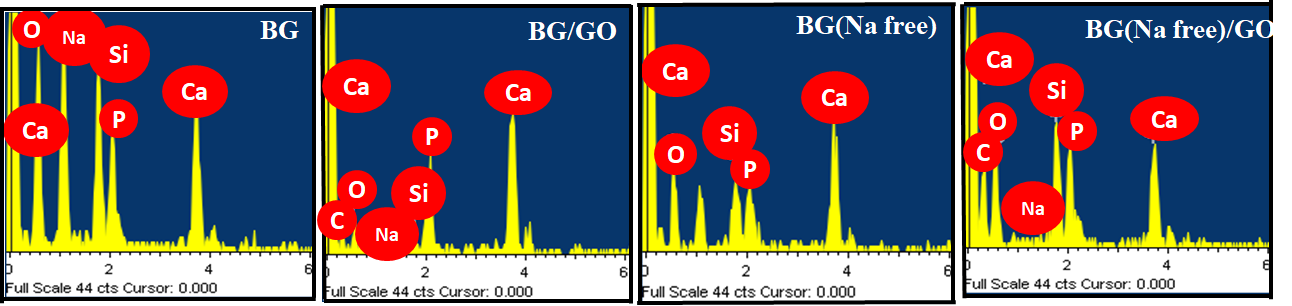


**Figure. S5.** EDAX spectrum of BG, BG (Na-free) and BG/GO, BG (Na-free)/GO nanocomposites before mineralization.

**Table S2.** Elemental Quantification of BG, BG (Na-free) and BG/GO, BG (Na-free)/GO nanocomposite.

| **Elements** | **Atomic (%)**  **(Before Mineralization)** | | | | **Atomic (%)**  **(After Mineralization)** | | | | |
| --- | --- | --- | --- | --- | --- | --- | --- | --- | --- |
|  | **BG** | **BG/GO** | **BG**  **(Na-free)** | **BG (Na-free)/GO** | **BG** | **BG/GO** | **BG (Na-free)** | **BG (Na-free)/GO** | **GO** |
| Si | 7.81 | 1.84 | 5.88 | 3.78 | 3.57 | 0.70 | 11.36 | 6.53 |  |
| Ca | 6.09 | 14.91 | 10.72 | 3.91 | 87.35 | 17.95 | 60.59 | 5.39 |  |
| P | 4.93 | 6.94 | 4.98 | 3.28 | 7.78 | 6.49 | 28.05 | 3.93 |  |
| C | - | 41.05 | - | 46.26 | - | 28.50 | - | 34.81 | 72.86 |
| O | 63.97 | 34.57 | 78.41 | 42.82 | - | 45.92 | - | 49.33 | 27.14 |
| Na | 17.20 | 0.69 | - | -0.06 | 1.30 | - | - | - |  |

**Table S3.** Determination of elemental peaks from XPS spectra.

| **Peaks** | **Binding**  **Energy (eV)** | **Chemical Bond** | **Peaks** | **Binding Energy (eV)** | **Chemical Bond** |
| --- | --- | --- | --- | --- | --- |
| **Si 2p** | 104.67 | SiO_2_ | **C 1s** | 286.92 | C-O |
|  | 101.53 | Si_3_N_4_ |  | 285.65 | C-OH |
|  | 101.3 | Si 2p_3/2_ |  | 284.77 | Sp^2^ C |
| **P 2p** | 134.6 | P 2p_3/2_ | **O 1s** | 532.7 | O-Si |
|  | 133.1 | PO_3_ |  | 531.3 | O-C |
|  | 131.79 | P-C |  | 535.6 | Si-O-Si |
|  | 1073.4 | Na^+^ |  | 532.82 | BO (Bridging Oxygen) |
| **Ca 2p** | 350.97 | Ca 2p_1/2_ |  | 532.93 | C=O, C-O-C |
|  | 348.2 | CaCO_3_ |  | 532.7 | O-Si |
|  | 347.15 | Ca(PO_4_)_2_ | **Na 1s** | 1072 | Na-O, Na-O-Si |
|  | 347 | Ca 2p_3/2_ |  |  |  |
| **C 1s** | 290 | C=O |  |  |  |
|  | 286.4 | C-O-C |  |  |  |
|  | 284.7 | C-C |  |  |  |

**1.4 Raman Spectra analysis**


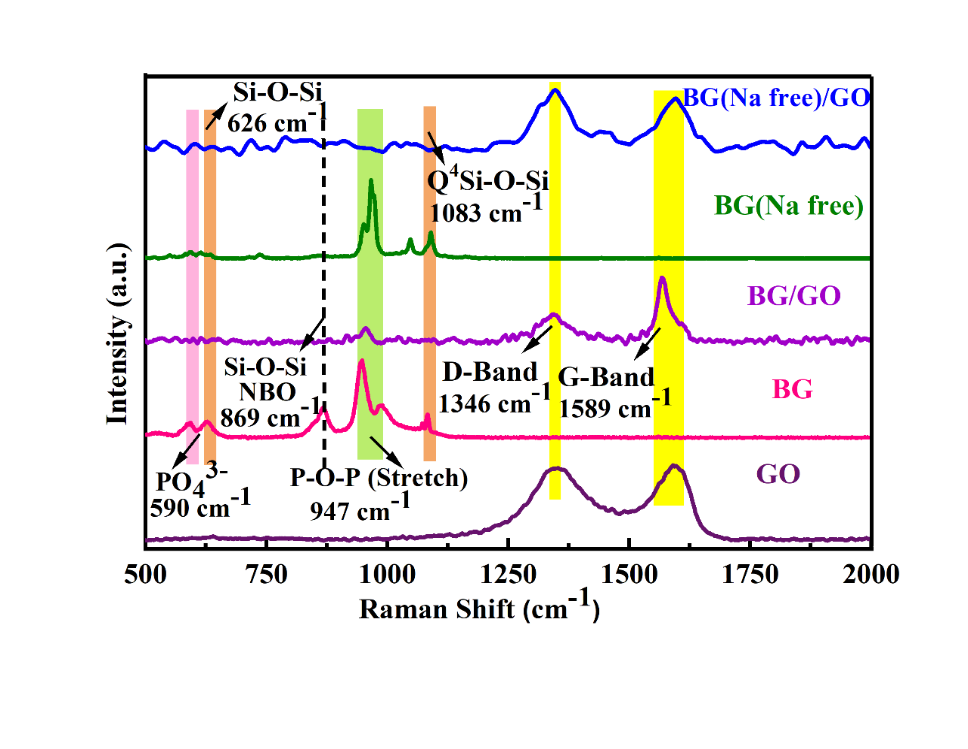


**Figure S6.** Raman spectra of BG, BG (Na-free), GO, and BG/GO, BG (Na-free)/GO nanocomposites.

**1.5 FTIR Analysis**


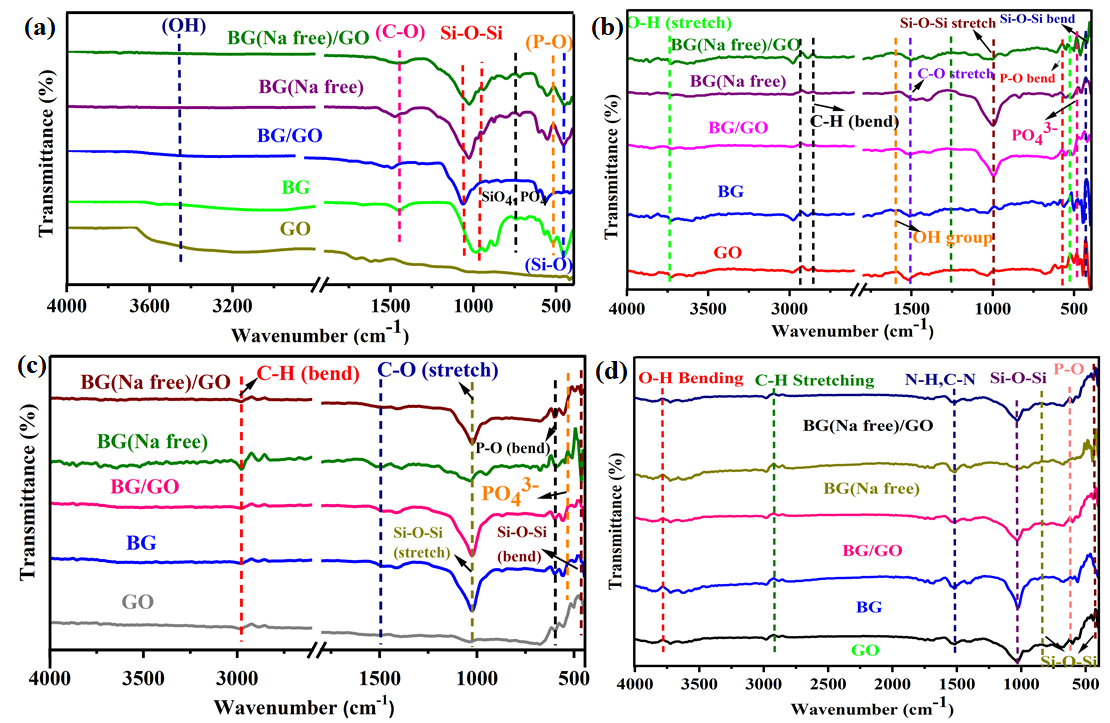


**Figure S7.**  FTIR band position of BG, BG (Na-free), GO and BG/GO, BG (Na-free)/GO nanocomposites; (a) Before Mineralization. After Mineralization; (b) 1^st^ day, (c) 14^th^ day, (d) 28^th^ day.

**Table. S4.** Determination of functional groups from FTIR spectra after mineralization.

| **Sample** |  | **After Mineralization**  **(Wavenumber cm^-1^)** | | | **Functional Group** | **Ref.** |
| --- | --- | --- | --- | --- | --- | --- |
|  | Before | (1^st^ D) | (14^th^ D) | (28^th^ D) |  | |
| GO | 3452 | 3738 | 3975 | 3775 | Free OH group | 1 |
|  |  | 2927 |  | 2919 | C-H bending | 2 |
| BG | 1445 | 1515 | 1492 | 1514 | C-H, C-O stretching,  Residual Carbonate group | 3 |
|  | 517 | 571 | 597 | -  - | P-O bending (crystal) | 4 |
|  | 453 | 449 | 465 |  | Si-O-Si bending | 5 |
| BG/GO | 1063 | 1026 | 1026 | 1032 | Si-O-Si anti-symmetric stretching | 6 |
| BG (Na-free) | 738 | - | - |  | SiO_4_, PO_4_^3-^ | 7 |
| BG (Na-free)/GO | - | | | 810 | Si-O-Si symmetric stretching | 8 |
|  | - | 679 | 602 | 622 | P-O bending (amorphous) | 9,10 |
|  | - | | | 1527 | N-H bending, C-N stretching (in-plane) | 11 |

**1.6 Thermal Studies**


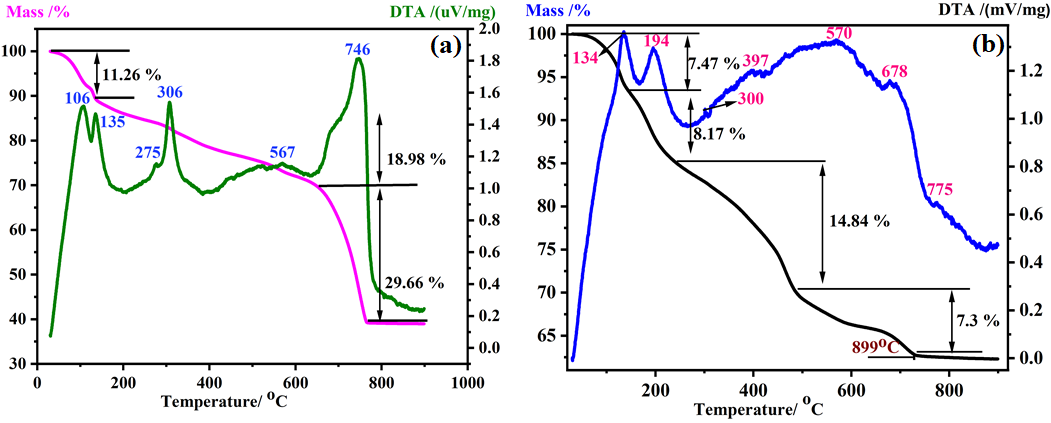


**Figure S8.** TG and DTA results of (a) BG, and (b) BG (Na-free) before annealing.

**1.7 Mechanical Stability Analysis**


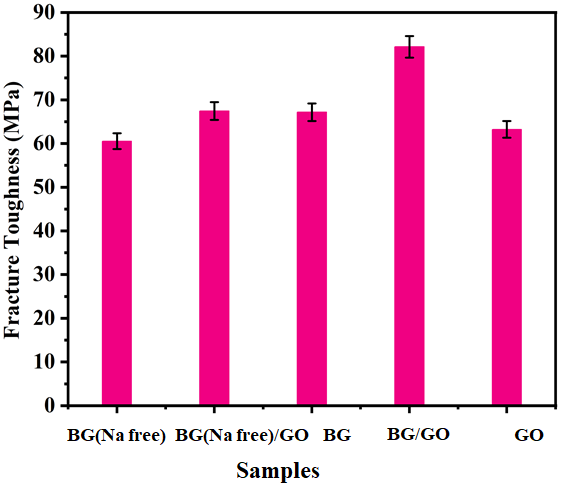


**Figure. S9.** Vickers hardness measurements for BG, BG (Na-free), GO and BG/GO, BG (Na-free)/GO nanocomposites.


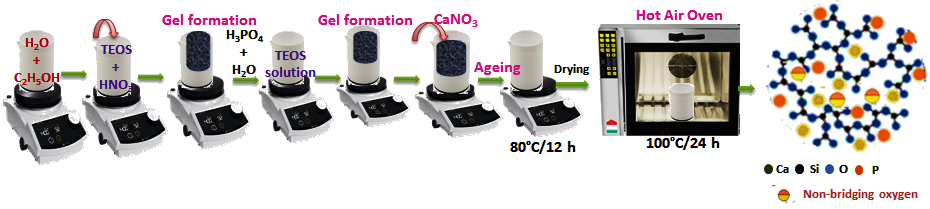


**Figure. S10.** Preparation of 45S5 BG by Sol-gel Method.


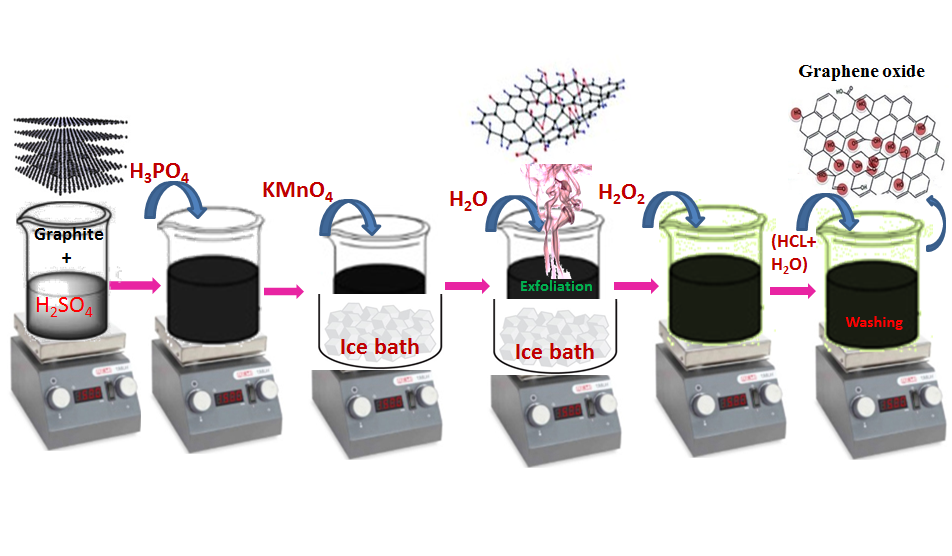


**Figure. S11.** Preparation of GO by Improved Hammers Method.


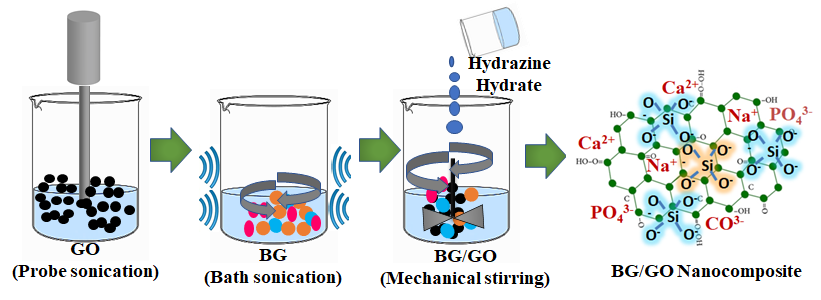


**Figure S12.** Schematic representation of the preparation of BG/GO nanocomposites.

**References**

1. Youning, G., Delong, L., Qiang, F. & Chunxu P. Influence of graphene microstructures on electrochemical performance for supercapacitors. *Prog. Nat. Sci.: Mater. Int.* **25**, 379-385 (2015). DOI: [10.1016/j.pnsc.2015.10.004](http://dx.doi.org/10.1016%2Fj.pnsc.2015.10.004).
2. Mendes, L. C., Ribeiro, G. L. & Marques, R. C. In Situ Hydroxyapatite Synthesis: Influence of Collagen on Its Structural and Morphological Characteristic. *Mat. Sci. Appl.* **3***,* 580-586 (2012). DOI: [10.4236/msa.2012.38083](http://dx.doi.org/10.4236/msa.2012.38083).
3. Mehdipour, M., Afshar, A. & Mohebali, M. Electrophoretic deposition of bioactive glass coating on 316L stainless steel and electrochemical behavior study. *Appl. Surf. Sci.* **258***,* 9832–9839 (2012). DOI:[10.1016/j.apsusc.2012.06.038](http://dx.doi.org/10.1016/j.apsusc.2012.06.038).
4. Faure, J., Drevet, R., Lemelle, A., Ben Jaber, N., Tara, A. & El Btaouri, H., Benhayoune, H. A new sol-gel synthesis of 45S5 bioactive glass using an organic acid as catalyst. *Mater. Sci. Eng. C* **47**, 407-412 (2015). DOI: 1[0.1016/j.msec.2014.11.045](https://doi.org/10.1016/j.msec.2014.11.045).
5. Vikash, K. V., Sampath Kumar, A., Akher, A., Sunil Prasad., Pradeep, S., Sarada Prasanna, M., Md Ershad., Saryoo Prasad, S. & Ram, P. Assessment of nickel oxide substituted bioactive glass-ceramic on in vitro bioactivity and mechanical properties. *Bol. Soc. Esp. Ceram. Vidr.* **55,** 228-238 (2016). DOI:[10.1016/j.bsecv.2016.09.005](http://dx.doi.org/10.1016/j.bsecv.2016.09.005).
6. Bargavi, P., Chitra, S., Durgalakshmi, D., Rajashree, P. & Balakumar, S. Effect of Titania Concentration in Bioglass/TiO_2_ Nanostructures and Its *In Vitro* Biological Property Assessment. *J. Nanosci. Nanotechnol.* **18**, 4746-4754 (2018). DOI: [10.1166/jnn.2018.15340](http://dx.doi.org/10.1166/jnn.2018.15340).
7. Bui, X. V. & Dang, T. H. Bioactive glass 58S prepared using an innovation sol-gel process. *Process Appl. Ceram.* **13**, 98–103 (2019). DOI: [10.2298/PAC1901098B](http://dx.doi.org/10.2298/PAC1901098B).
8. Tuan, T. A., Guseva, E. V., Tien, N. A., Dat, H. T. & Vuong, B. X. Simple and Acid-Free Hydrothermal Synthesis of Bioactive Glass 58SiO_2_-33CaO-9P_2_O_5_ (wt%). *Crystals* **11***,* 283 (2021). DOI: [10.3390/cryst11030283](http://dx.doi.org/10.3390/cryst11030283).
9. Wei, L., Hui, W., Yaping, D., Scheithauer, E. C., Goudouri, O. M., Grunewald, A., Detsch, R., Agarwal, S. & Boccaccini, A. R. Antibacterial 45S5 based scaffolds reinforced with genipin cross-linked gelatin for bone tissue engineering. *J. Mater. Chem. B* **3***,* 3367-3378 (2015). DOI: [10.1039/C5TB00044K](https://doi.org/10.1039/C5TB00044K).
10. Aguilar-Reyes, E. A., Leon-Patino, C. A., Villicana-Molina, E., Macias-Andresa, V. I. & Lefebvre, L. P. Processing and *in vitro* bioactivity of high-strength 45S5 glass-ceramic scaffolds for bone regeneration. *Ceram. Inter.* **43,** 6868-6875 (2017). DOI: [10.1016/j.ceramint.2017.02.107](http://dx.doi.org/10.1016/j.ceramint.2017.02.107).
11. Nazia, B., Suzi Salwah, J., Hatijah, B., Sharifah, A. & Dagaci Muhammad, Z. XRD and FTIR Study of A&B Type Carbonated Hydroxyapatite Extracted from Bovine Bone. *AIP Conf. Proc.* **2068***,* 020100 (2019). DOI: [10.1063/1.5089399](https://doi.org/10.1063/1.5089399).
